# β-Sitosterol targets Trx/Trx1 reductase to induce apoptosis in A549 cells via ROS mediated mitochondrial dysregulation and p53 activation

**DOI:** 10.1038/s41598-018-20311-6

**Published:** 2018-02-01

**Authors:** Tamilselvam Rajavel, Pandian Packiyaraj, Venkatesan Suryanarayanan, Sanjeev Kumar Singh, Kandasamy Ruckmani, Kasi Pandima Devi

**Affiliations:** 1 Department of Biotechnology, Science Campus, Alagappa University, Karaikudi-630 003, Tamil Nadu, India; 2 Computer Aided Drug Design and Molecular Modeling Lab, Department of Bioinformatics, Alagappa University, Karaikudi- 630003, Tamil Nadu, India; 3 National Facility for Drug Development (NFDD) for Academia, Pharmaceutical and Allied Industries, Bharathidasan, Institute of Technology, Anna University, Tiruchirappalli, 620024, Tamil Nadu, India

## Abstract

β-Sitosterol (BS), a major bioactive constituent present in plants and vegetables has shown potent anticancer effect against many human cancer cells, but the underlying mechanism remain elusive on NSCLC cancers. We found that BS significantly inhibited the growth of A549 cells without harming normal human lung and PBMC cells. Further, BS treatment triggered apoptosis via ROS mediated mitochondrial dysregulation as evidenced by caspase-3 & 9 activation, Annexin-V/PI positive cells, PARP inactivation, loss of MMP, Bcl-2-Bax ratio alteration and cytochrome c release. Moreover, generation of ROS species and subsequent DNA stand break were found upon BS treatment which was reversed by addition of ROS scavenger (NAC). Indeed BS treatment increased p53 expression and its phosphorylation at Ser15, while silencing the p53 expression by pifithrin-α, BS induced apoptosis was reduced in A549 cells. Furthermore, BS induced apoptosis was also observed in NCI-H460 cells (p53 wild) but not in the NCI-H23 cells (p53 mutant). Down-regulation of Trx/Trx1 reductase contributed to the BS induced ROS accumulation and mitochondrial mediated apoptotic cell death in A549 and NCI-H460 cells. Taken together, our findings provide evidence for the novel anti-cancer mechanism of BS which could be developed as a promising chemotherapeutic drug against NSCLC cancers.

## Introduction

Non-small cell lung cancer (NSCLC) is one of the common aggressive malignant tumor accounting for about 85% of human lung cancers^1^. Global statistical report published in 2012 revealed that, NSCLC related deaths are the major heath burden issue in both well and less developed nations^2^. Though smoking and tobacco consumption are considered as the major risk factors, NSCLC cases are also prevalent in non-smokers. In spite of the tremendous advancements made in the recent years in the different areas of cancer research, especially in diagnosis, chemotherapy, targeted therapy and radiation therapy, the major factors which limit the treatment outcome are, poor prognosis of the disease and late diagnosis. In addition, the effects of classical chemotherapeutical anticancer agents are also frequently attenuated due to the development of drug resistance. As a result, the intake of higher dose of the drugs are incapable of improving the treatment efficiency, rather causes adverse side effects in non-targeted tissues. This has actually resulted in insistent need to explore new molecules which could combat NSCLC with potential anticancer efficiency along with significant safety and devoid of toxicity.

In recent years, there have been growing interests on assessing the potential of natural products against human cancers. Among the different sources of drugs, plant derived phytochemicals have shown promising effect in both preclinical and clinical models^3,4^. Phytosterols, which are the plant sterols are one among the phytochemicals that has shown potential anticancer effect along with exhibiting good safety profile^5–9^. β-Sitosterol (BS) is the plant sterol that is most abundantly present in plants and structurally similar to cholesterol, except in the addition of ethyl group. It is consumed from the various dietary sources like herbal products, soy products, flax seed, vegetable oil, peanuts and peanut products^10,11^, with a daily average consumption rate of about 160–400 mg^12^. Numerous studies have evidenced that the anticancer effect of BS was associated with the induction of apoptosis through blockade of multiple cell signaling mechanisms^10^. For instance, BS activates apoptosis in leukemic cancer cell lines by inducing G2/M arrest. Molecular studies have shown that BS induces endoreduplication in U937 and HL60 cells by promoting spindle microtubule dynamics through the Bcl-2 and PI3K/Akt signaling pathways^11^. BS is also effective against breast, prostate, stomach and colon tumors by targeting different signaling pathways which induces apoptosis^12–16^. However the effect of BS on NSCLC largely remains unknown and the mechanism by which BS stimulates apoptosis requires further investigation.

In this study we have demonstrated for the first time that BS is effective against human NSCLC cells and the investigation of the molecular mechanism has revealed that BS promotes apoptotic cell death in A549 and NCI-H460 cells through ROS accumulation and loss of ΔΨ m via p53 activation. Further, our data revealed that BS inhibits the protein expression of Trx/TrxR1, which in turn triggers ROS accumulation in A549 and NCI-H460 cells and activation of apoptotic cell death.

## Results

### BS significantly inhibits the growth of A549 cells without harming normal cells

**Figure 1:**
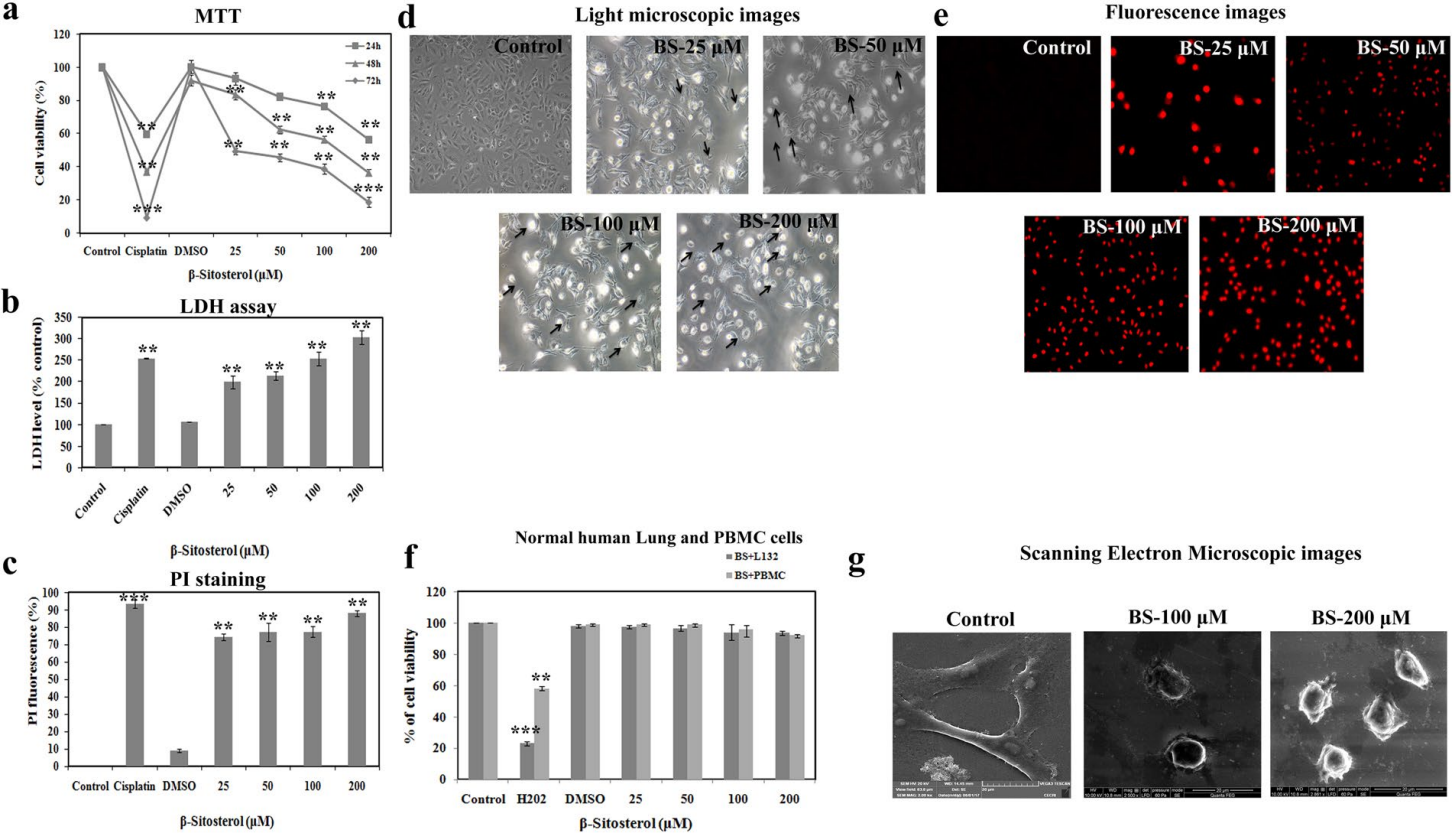
β-Sitosterol inhibits the cell proliferation of A549 cells and induces cell membrane damage. (**a**) Cytotoxic effect of BS on A549 cells was measured by MTT assay. Cells were treated with increasing concentration of BS for different time points 24, 48 and 72 h. (**b**) LDH leakage in A549 cells upon BS treatments after 72 h incubation. (**c**) Propidium iodide stained A549 cells after 72 h incubation with BS treatment, which indicated that BS treatment caused severe cell membrane damage. (**d**) Phase contrast microscopic images of BS treated A549 cells which appeared in cell shrinkage, reduced cell density and membrane blebbing. (**e**) Representative images of PI stained A549 cells upon BS treatment after 72 h. (**f**) Non-toxic effect of BS on non-malignant lung (L132 cell line) and PBMC cells. (**g**) Scanning Electron Microscopic images of BS treated cells with indicated concentrations that showed severe cell surface damages with loss of cell microvilli. The values are expressed as means ± S.D. of triplicates. *P < 0.05, **P < 0.01, ***P < 0.0001, significantly different compared with control.

Initially, we assessed the anti-proliferative effect of BS on A549 cells with different concentrations and at different time points (24, 48 and 72 h) using three different experiments. The MTT results (Fig. 1a) revealed that, BS significantly affected the growth of A549 cells in a concentration and time different manner. However, strong growth inhibition was found after 72 h time point with the IC_50_ value of 24.7 μM. In addition, the results of LDH (Lactose dehydrogenase) leakage assessment showed that (Fig. 1b), the release of LDH was concentration dependent upon BS treatment on A549 cells. It also indicated the loss of cell viability and damage in cell membrane during BS exposure. Further, fluorescence images of the cells stained with propidium iodide (PI) revealed that increasing number of PI positive cells were observed during treatment with BS. These results also substantiated that cell membrane damage and loss of cell viability was induced upon BS treatment after 72 h of incubation time. However, BS did not affect the growth and viability of normal human lung and PBMC cells following BS exposure at 72 h (Fig. 1f). Taken together, our results clearly confirmed that BS affects the growth of A549 cells by a time and dose dependent manner without affecting the cell proliferation of non-malignant human cells.

We further monitored the morphological changes induced by different concentrations of BS in A549 cells under phase contrast microscope. In the absence of BS, A549 cells appeared with typical epithelial morphology with high density of cell populations, whereas after 72 h of BS treatment, cell shrinkage, cell elongation and appearance of apoptotic bodies with reduced cell populations was observed. In addition, the morphological changes were also examined in scanning electron microscope. SEM analysis showed (Fig. 1g) remarkable damage in the cell surface of A549 in the presence of BS, which confirmed that the anti-proliferative effect of BS has been associated with cell membrane damage followed by cell death. Overall, the results of cytotoxic experiments and observance of morphological changes confirmed that the anticancer action of BS could be associated with apoptotic mode of cell death in A549 cells.

### BS induces cell cycle arrest at Sub-G1 phase

**Figure 2:**
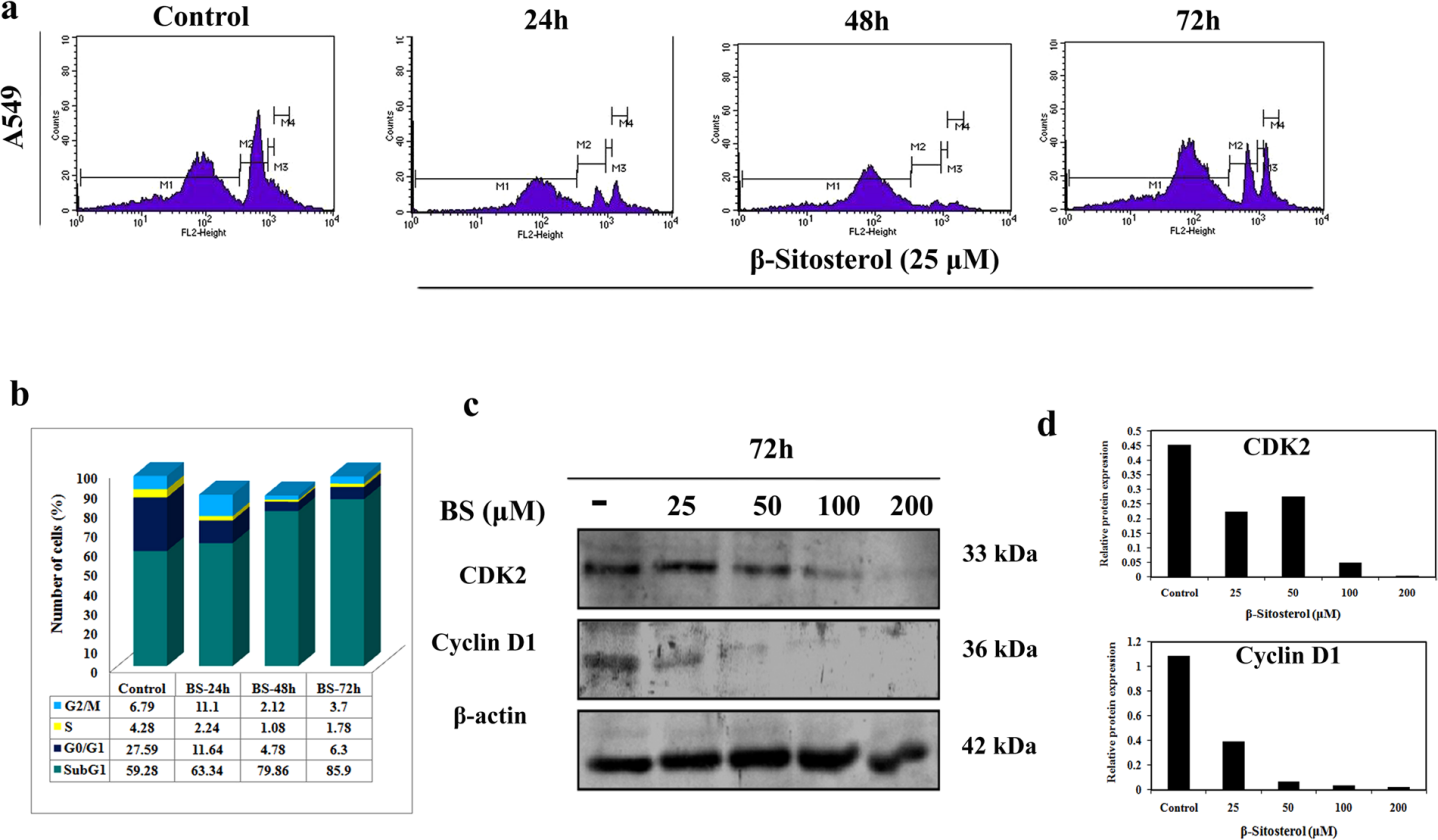
BS induced cell cycle arrest at sub-G1 phase and modulation of cell cycle proteins in A549 cells. (**a**) Cell cycle distribution of A549 cells under BS treated condition after different time points of BS exposure at indicated concentration. BS treatment increased the cells in Sub-G1 population (**b**) Histogram of cell cycle distribution in A549 cells. (**c**) Expression of cyclin d and cdk2 upon BS treatment on A549 cells by western blot analysis. (**d**) Relative protein expression of cyclin d and cdk 2 after 72 h of BS treatment was done using ImageJ analysis. The gel blots were cropped from different gels and the full length blots are given in the supplementary file.

To determine whether the anticancer action of BS has any impact on cell cycle, the cell cycle distribution was analyzed by flow cytometry method. A549 cells was treated with the IC_50_ concentration of BS (25 μM) for different time points and DNA content was measured using PI staining followed by flow cytometry analysis. As shown in Fig. 2a, a time dependent increase of Sub-G1 population cells was observed which peaked at 72 h upon BS treatment. Accumulation of cells in Sub-G1 population phase is a well known marker for cells undergoing apoptotic mode of cell death. Hence, the cell cycle analysis positively indicated that the anti-proliferative action of BS could be associated with apoptotic mode of cell death in A549 cells. We also examined changes in the expression of the major cell cycle regulators Cyclin-D1 and CDK-2 by western blot analysis, after 72 h treatment of BS at different concentrations. The results showed that (Fig. 2c) the expression of both Cyclin-D1 and CDK-2 was dose dependently reduced by BS treatment. Maximum concentration of BS displayed significant reduction in the expression of both Cyclin D1 and CDK2. Taken together, down-regulation of Cyclin-D1, CDK-2 and increased Sub-G1 cell populations confirmed that the anticancer action of BS has been associated with cell cycle arrest and apoptosis.

### BS triggers apoptosis through intrinsic caspase pathway

**Figure 3:**
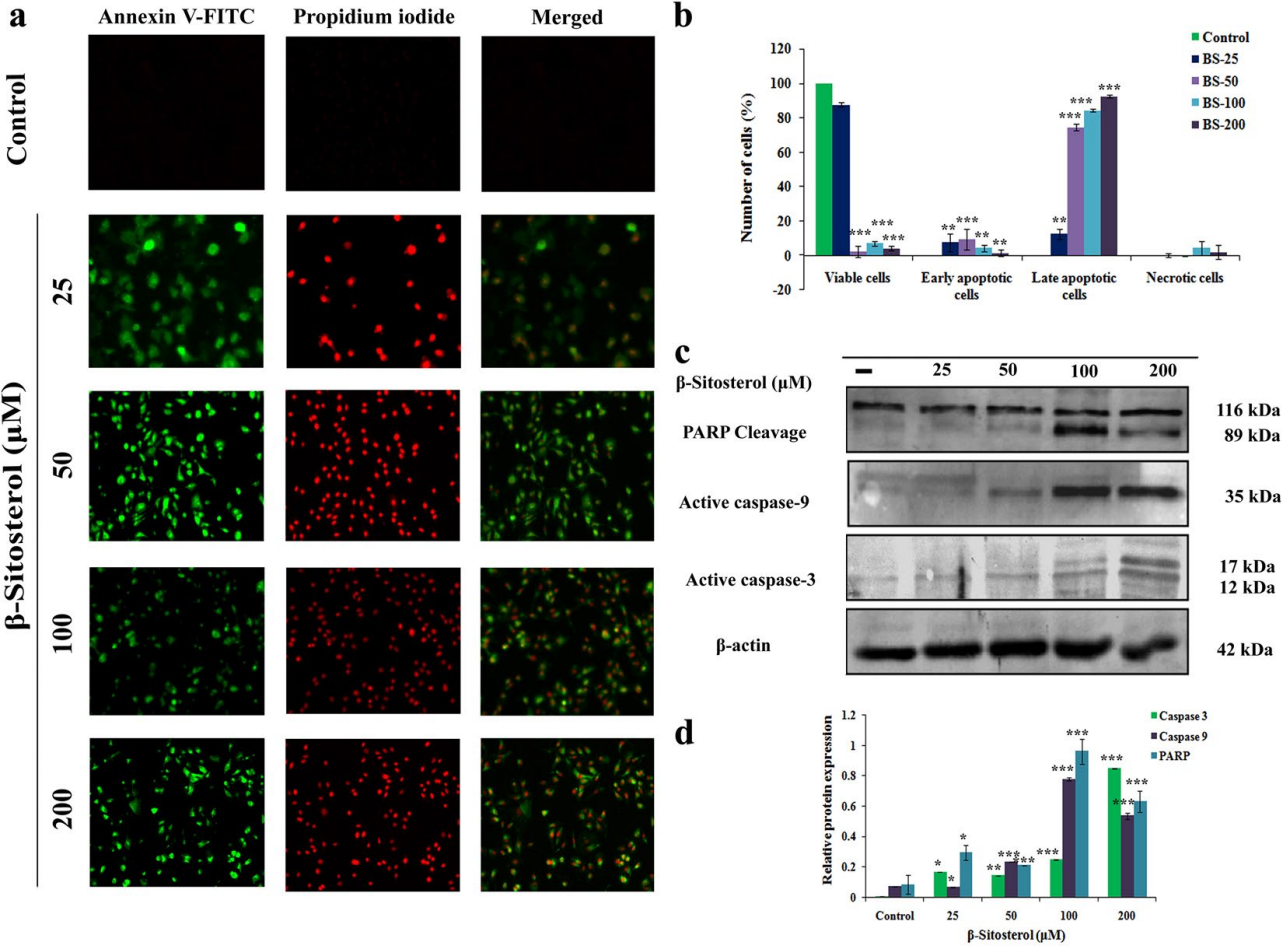
BS triggered caspase mediated apoptotic pathway on A549 cells. (**a**) BS induced increasing number of apoptotic cells at indicated concentrations of BS at 72 h, which were imaged by using Annexin-V/FITC and PI dual staining. (**b**) Quantitative measurement of apoptotic and necrotic cells upon BS treatment in A549 cells after 72 h treatment. (**c**) Expression of apoptosis related proteins under BS induced condition on A549 cells were analysed by western blotting. Significant up-regulation of caspase-3, caspase-9 and PARP cleavage indicated activation of apoptotic pathway upon BS treatment. (**e**) Relative protein expression of apoptotic proteins upon different concentrations of BS treated A549 cells. The gel blots were cropped from different gels and the full length blots are given in the supplementary file. Quantification was done using ImageJ analysis. *P < 0.05, **P < 0.01, ***P < 0.0001, significantly different when compared with control.

To confirm the effect of BS on apoptosis, we initially performed Annexin-V/FITC and PI staining followed by fluorescent microscopic analysis. A549 cells were treated with various concentrations of BS for 72 h and the percentages of apoptotic cells were quantitatively measured by dual staining of Annexin V/FITC and PI. A significant increase in early apoptotic cells and few late apoptotic cells were observed upon BS (25 μM) exposure (Fig. 3a). However, on increasing the concentration of BS, a dose dependent increase of apoptotic cells were observed without causing the formation of any necrotic cells. Altogether the results suggest that BS may promote apoptotic mode of cell death on A549 cells by a dose dependent passion. Hence, in an attempt to understand the molecular events associated with BS induced apoptosis, the expression level of the apoptotic proteins were confirmed by western blotting. The expression of the active caspases (caspase-3 and caspase-9) was first determined in the cells treated with different concentrations of BS at 72 h exposure time point. A significant elevation of active caspase-3 (12 & 17 kDa) was observed during treatment with maximum concentration of BS (200 μM) (Fig. 3c). Similarly, BS treatment also displayed strong up-regulation of active caspase-9 (35 kDa), which indicated that BS induced apoptosis occurred via intrinsic apoptotic pathway in A549 cells. Moreover, up-regulation of effective caspases resulted in cleavage or inactivation of PARP protein, which serve as a biochemical marker for the cells undergoing apoptosis^17^. Inactivation of PARP protein was prominently observed in A549 cells in all the concentration of BS and it was more significant at 200 μM of BS. Taken together, the results revealed that the anticancer action of BS was associated with activation of intrinsic mode of apoptotic cell death in A549 cells.

### BS induced apoptosis occurred via mitochondrial dysfunction

**Figure 4:**
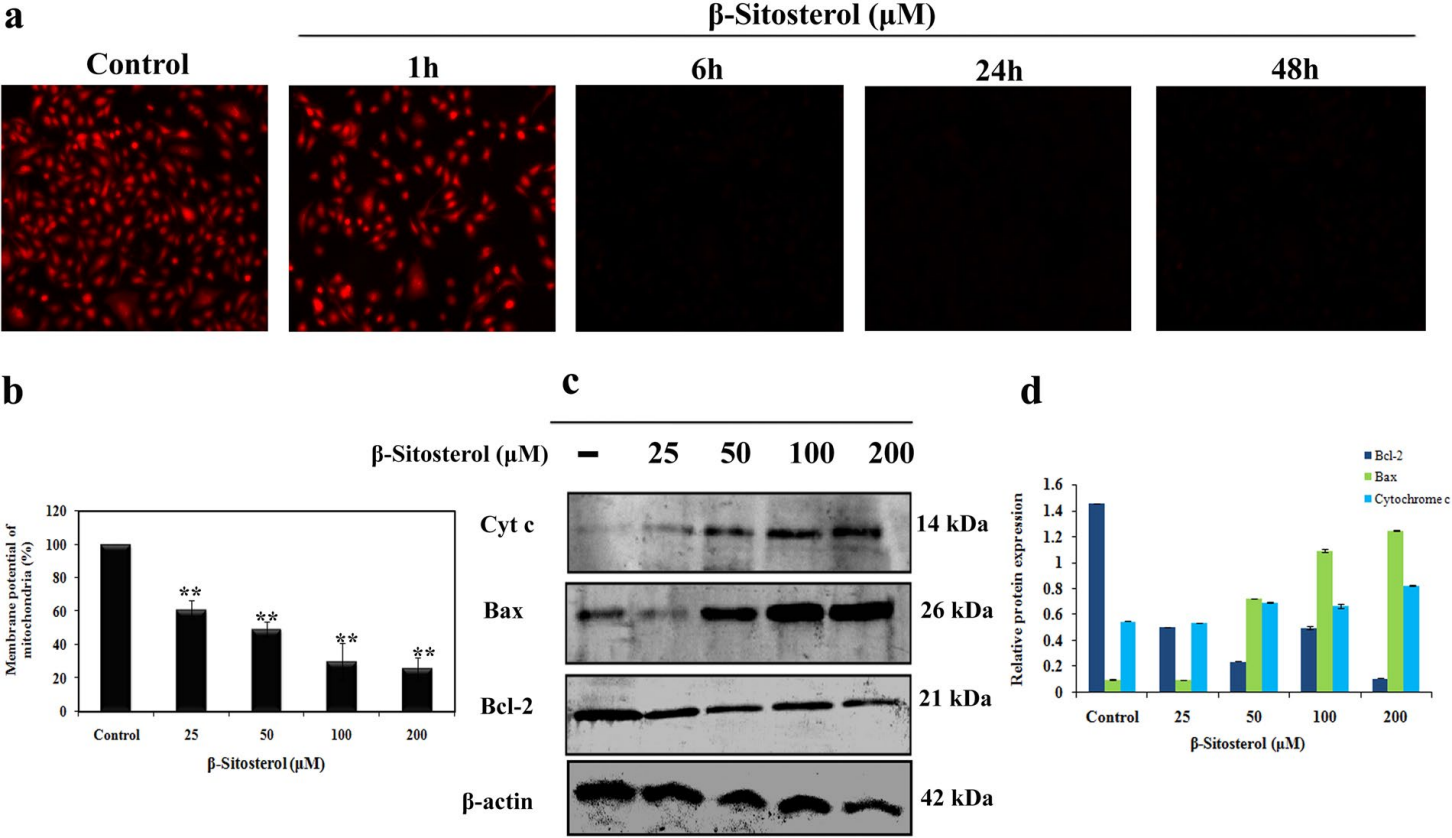
BS induced apoptosis occurred via mitochondrial membrane permeability loss and releases of apoptogenic factors. (**a**) Loss of ΔΨ m in A549 cells at different time points of BS treatment (25 μM) were imaged by Rhodamine 123 staining. (**b**) Fluorescence spectroscopy analysis of Rhodamine 123 stained BS treated cells. (**c**) Up-regulation of bax and cytochrome c was detected upon BS treatment, while significant down-regulation of bcl-2 was also observed after 72 h incubation. (**d**) Relative protein expression of bcl-2 family protein and cytochrome c release. The gel blots were cropped from different gels and the full length blots are given in the supplementary file. **P < 0.01 versus control.

Caspases are activated through either intrinsic or extrinsic apoptotic mode of pathways. However, activation of caspase-3 & 9 was observed in A549 cells following treatment with BS for 72 h, which strongly suggested involvement of mitochondria (Intrinsic) mediated apoptotic pathway. Moreover, Intrinsic apoptotic mode of cell death was activated only upon loss of mitochondrial membrane potential (ΔΨ m)^18^. Hence, we investigated whether the BS induced apoptosis occurred through loss of ΔΨ m. A549 cells were treated with IC_50_ concentration of BS for different time points and changes in ΔΨ m was monitored by using mitochondrial specific probe Rhodamine 123. Rho 123 is a cationic fluorescent dye that accumulates in the active mitochondria, however during the loss of ΔΨ m, the rho 123 gets eluted from the cells. Therefore the decrease in fluorescence indicates loss of ΔΨ m in drug treated or apoptotic conditions. Fluorescence images showed (Fig. 4a) that a time dependent loss of ΔΨ m was observed in BS treated cells and a remarkable disruption was observed at maximum time points (24 h & 48 h). On the other hand, the untreated A549 cells displayed higher intake of Rho 123 and exhibited strong fluorescence intensity. Quantitative analysis by fluorescence spectrophotometer analysis showed that BS induced a concentration dependent disruption of ΔΨ m after 72 h treatment of BS (Fig. 4b). These results clearly indicated that BS induced apoptosis was associated with loss of ΔΨ m and activation of intrinsic mode of apoptotic cell death in A549 cells. However, the disruption of mitochondrial membrane potential results in release of apoptogenic factors in the cytoplasm which can further execute the caspase cascade pathway^18^. Therefore we further investigated the key players involved in the mitochondrial mediated apoptosis by western blot analysis. Initially we determined the expression of cytochrome c, after 72 h exposure of BS in A549 cells. As shown in Fig. 4c, treatment of BS significantly induced the release of cytochrome c into the cell cytoplasm in a dose dependent manner. In addition, it is well established that Bcl-2 family members act as regulators of mitochondrial membrane permeability. Hence, we examined the expression level of pro-apoptotic and anti-apoptotic proteins upon BS treatment by western blotting. The results showed (Fig. 4c) that BS treatment significantly suppressed the oncogenic expression of anti-apoptotic protein bcl-2 and strongly increased the expression of pro-apoptotic protein bax in a dose dependent manner. Taken together, the results confirmed that BS activates mitochondria-mediated apoptosis in A549 cells and has been associated with down-regulation of Bcl-2/Bax expression ratio.

### ROS mediated DNA damage is involved in BS induced apoptosis

**Figure 5:**
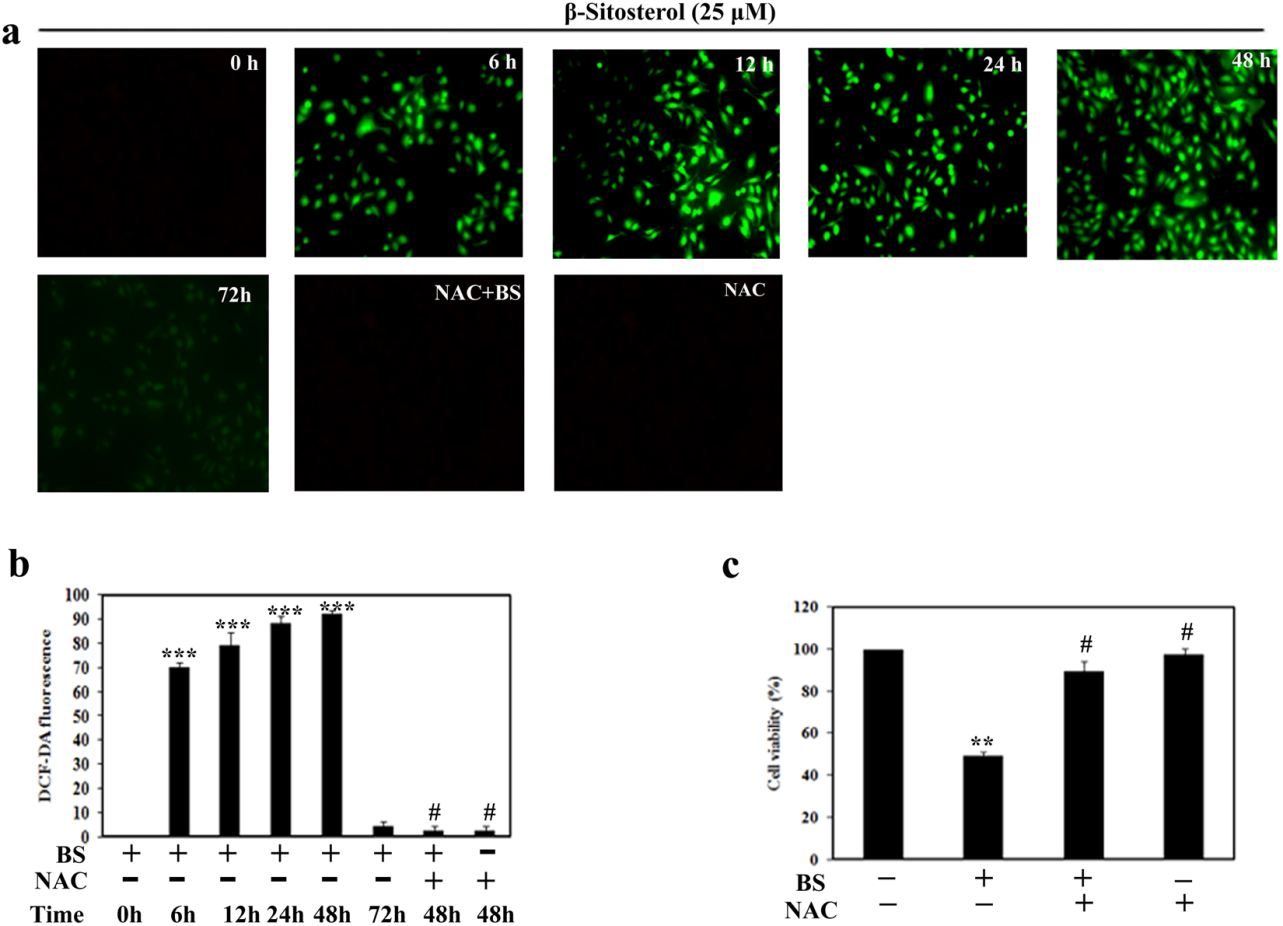
BS induced ROS dependent apoptosis in A549 cells. (**a**) Fluorescence images of DCF-DA stained BS treated (25 μM) A549 cells with indicated times at a 200× magnification. Cells were pretreated with or without 10 mM NAC, followed by 25 μM BS exposure for different time points. (**b**) DCF-DA fluorescence with and without NAC, upon BS treatment at various time intervals (0, 6, 12, 24 & 48 h). (**c**) Cell viability of A549 cells in the presence and absence of NAC after 48 h treatment of BS. *P < 0.05, **P < 0.01, ***P < 0.0001, significantly different when compared with control, ^#^P < 0.05 for BS versus NAC + BS.

ROS plays a significant role in apoptosis activation by many chemotherapeutical drugs and radiation treatments^19^. Indeed, cancer cells produce higher frequency of ROS generation than normal cells in their mitochondrial respiratory chain, which is an attractive strategy to selectively kill the cancer cells^20^. In the context of aberrant metabolism and signaling, tumor cells have well adapted in the imbalanced redox status through improved antioxidant capacity and oncogenic signaling pathways^21^. Due to its adaptive mechanism, the cancer cells become insensitive to even in high ROS levels. Importantly, observation of ΔΨ m loss and release of apoptogenic factors in A549 cells upon BS treatment, led us to investigate the role of ROS on BS induced mitochondrial mediated apoptosis. Initially we determined the ROS levels at different time points of BS treatment in A549 cells using the ROS specific fluorescent probe DCF-DA. As shown in Fig. 5a & b, both the fluorescence images and spectrofluorometric analysis depicted that generation of DCF fluorescence initiated at 6 h of BS treatment and it remarkably peaked at 12–48 h time point, followed by a decrease at 72 h. These results confirmed that BS induced apoptosis was associated with the ROS accumulation in A549 cells.

**Figure 6:**
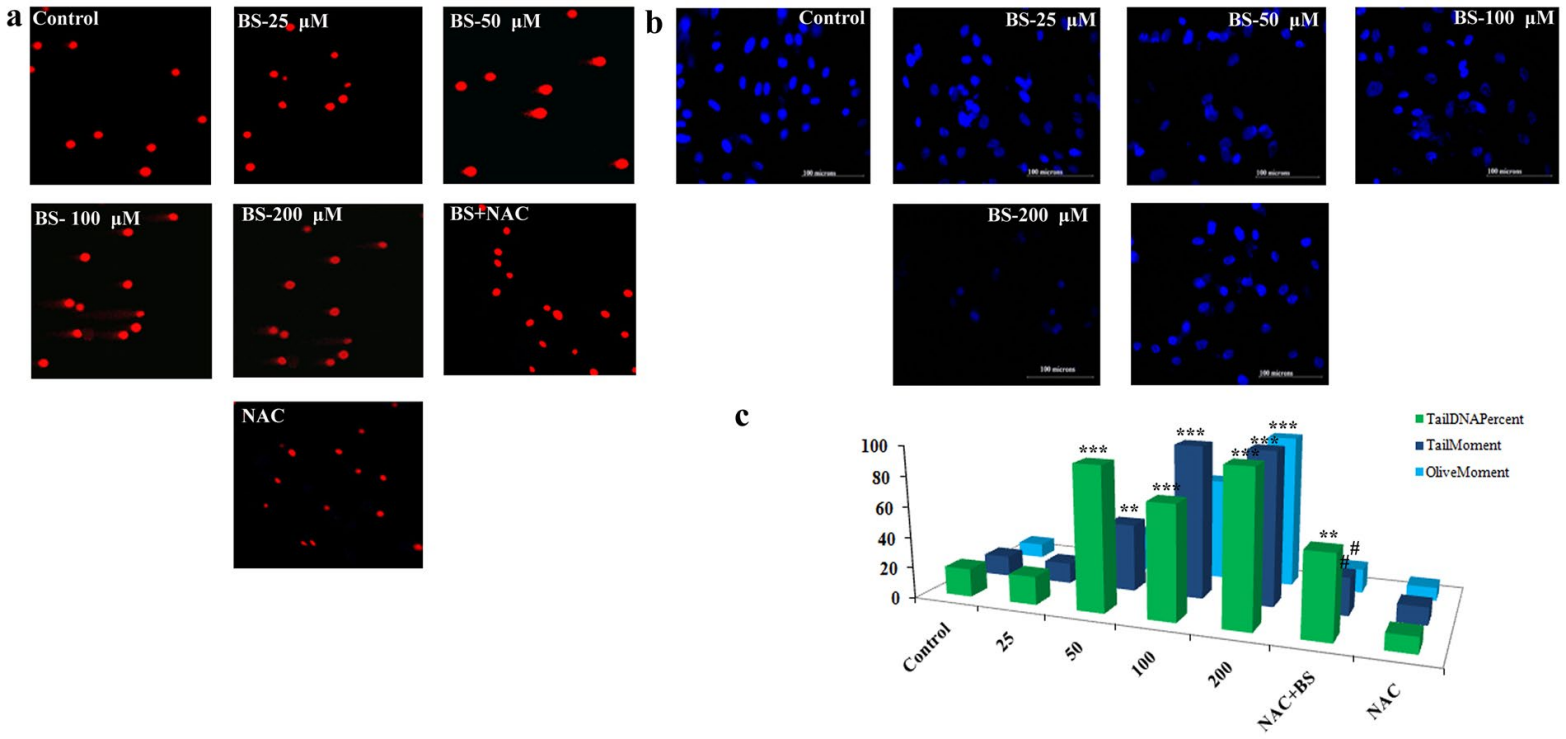
BS induced ROS and apoptosis associated with DNA damage in A549 cells. (**a**) Panel of fluorescence microscopic images showed comet like structure upon BS treatment after 72 h incubation time and this effect was reversed by addition of NAC (10 mM). (**b**) Nuclear morphological changes induced by BS were assessed by DAPI staining and visualized in fluorescence microscopy at a magnification of 200×. NAC reversed the nuclear morphological changes induced by BS. (**c**) Representative histogram of different comet factors on BS exposure was done using ImageJ software. *P < 0.05, **P < 0.01, ***P < 0.0001, significantly different when compared with control, ^#^P < 0.05 for BS versus NAC + BS.

To further confirm the role of BS in inducing ROS, we studied the ROS inducing efficacy of BS in the presence of the ROS scavenger NAC (N-acetylcysteine). Pretreatment of NAC significantly scavenged the BS induced ROS in A549 cells. Moreover, NAC treatment also affected the anti-proliferative property of BS against A549 cells. Therefore, it was clear that the generation of ROS plays a crucial role in BS induced apoptosis in A549 cells. In addition, ROS generation leads to macromolecular damage such as DNA strand break, increasing protein carbonyl content and lipid per-oxidation. Hence, we analyzed the classical marker DNA strand break (DSB) on A549 cells upon treatment of BS in the presence and absence of NAC by Comet assay. The results showed that (Fig. 6a) BS treatment caused severe DSBs in A549 cells and significant elevation of tail and olive movement was observed. Interestingly, the presence of NAC (10 mM) significantly abrogated the BS induced DNA damage and it disclosed that NAC protects the A549 cells from ROS. Moreover, we also examined the BS induced DNA damage by DAPI staining. As shown in Fig. 6b, BS treatment caused chromatin condensation and morphological alteration in the nucleus of A549 cells, which substantiated the results of Comet assay. Similarly, NAC treatment significantly reversed the BS induced DNA damages on A549 cells. Collectively, the results from Comet assay and DAPI staining clearly confirmed that the apoptotic effect of BS completely depends on ROS production in A549 cells.

### Involvement of p53 in BS induced apoptosis

**Figure 7:**
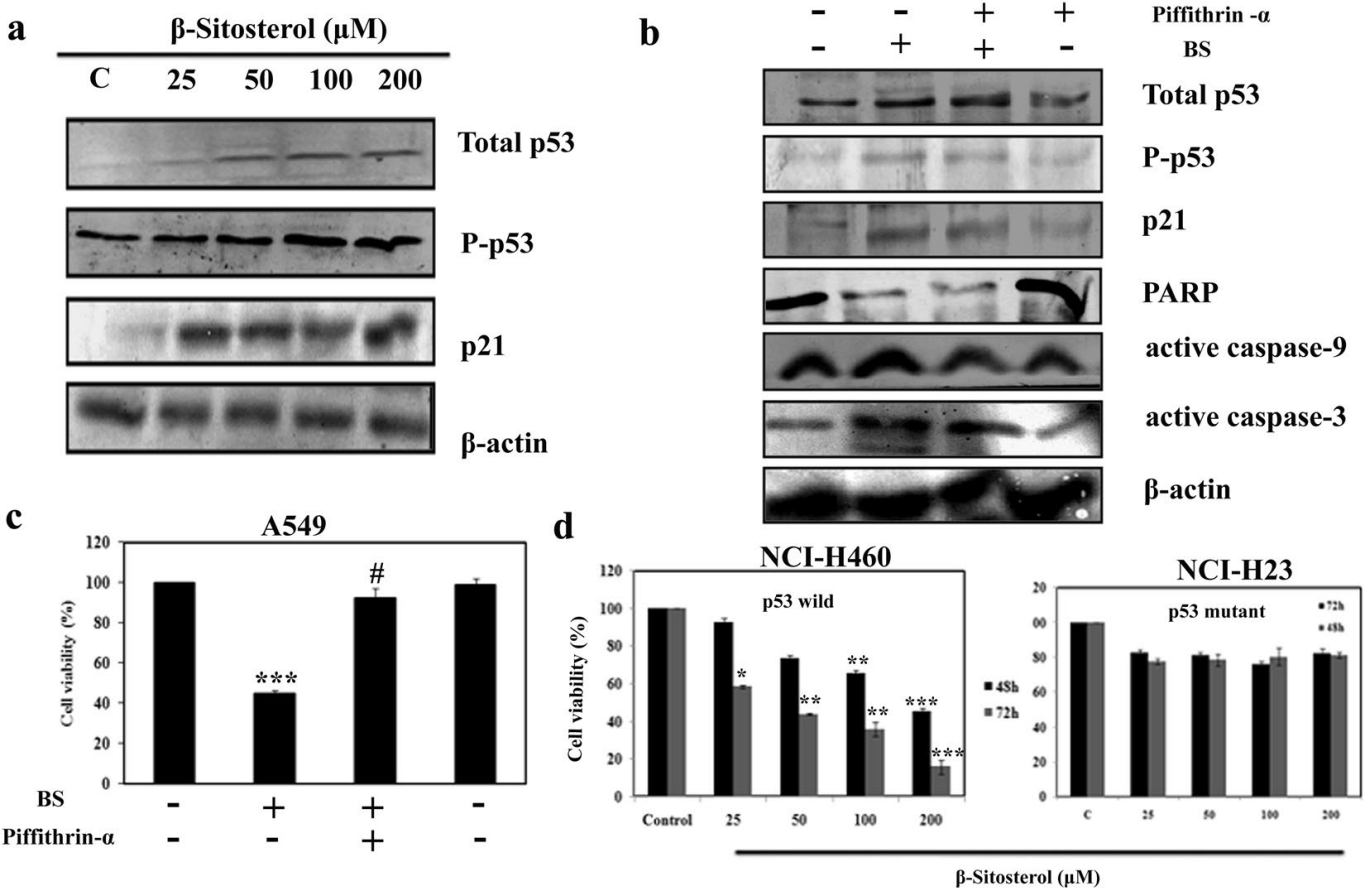
Excessive ROS production activates the p53 dependent pathway to induce the apoptosis on A549 cells. (**a**) The protein expression of p53, P-p53, p21were measured by western blotting. A549 cells were treated with varying concentrations of BS at 72 h and protein expressions were analyzed. (**b**) Protein expression of of p53, P-p53, p21, caspase-3, caspase-9, PARP in presence and absence of p53 inhibitor pifithrin-α at 1 μM. (**c**) Cell viability of A549 cells upon BS treatment in the presence and absence of pifithrin-α. (**d**) Cytotoxic effect of BS on p53 wild (NCI-H460) and mutant (NCI-H23) NSCLC cancer cell lines were assessed by MTT assay at 72 h time point. The gel blots were cropped from different gels and the full length blots are given in the supplementary file. *P < 0.05, **P < 0.01, ***P < 0.0001 versus control, ^#^P < 0.05 for BS versus Pifithrin-α + BS.

Activation of p53 can occurs in response to a diverse number of cellular stress including DNA damage, cell death, oxidative stress and hypoxia^22^. Apoptosis induction and DNA damage by ROS generation indicates the possibility of p53 involvement in BS induced apoptotic mitochondrial deregulation^23,24^. Hence, we employed western blotting to understand whether p53 plays any role in BS induced ROS mediated mitochondrial apoptosis. We determined the total protein expression of p53 and pSer15-p53 (p53 phosphorylation at Ser 15) during treatment with different concentrations of BS for 72 h incubation time. The results showed (Fig. 7a) strong up-regulation of p53 upon BS treatment and increased expression of pSer15-p53. Concomitantly, significant elevation of the p53 down-stream target protein p21^Cip1^ was also observed. P53 is the major tumor suppressor protein which is activated during DNA damage, apoptosis, cell cycle blockage and so on. Hence activation of p53 and p21 proteins by BS substantiated the role of BS in inducing DNA damage and cell cycle arrest.

**Figure 8:**
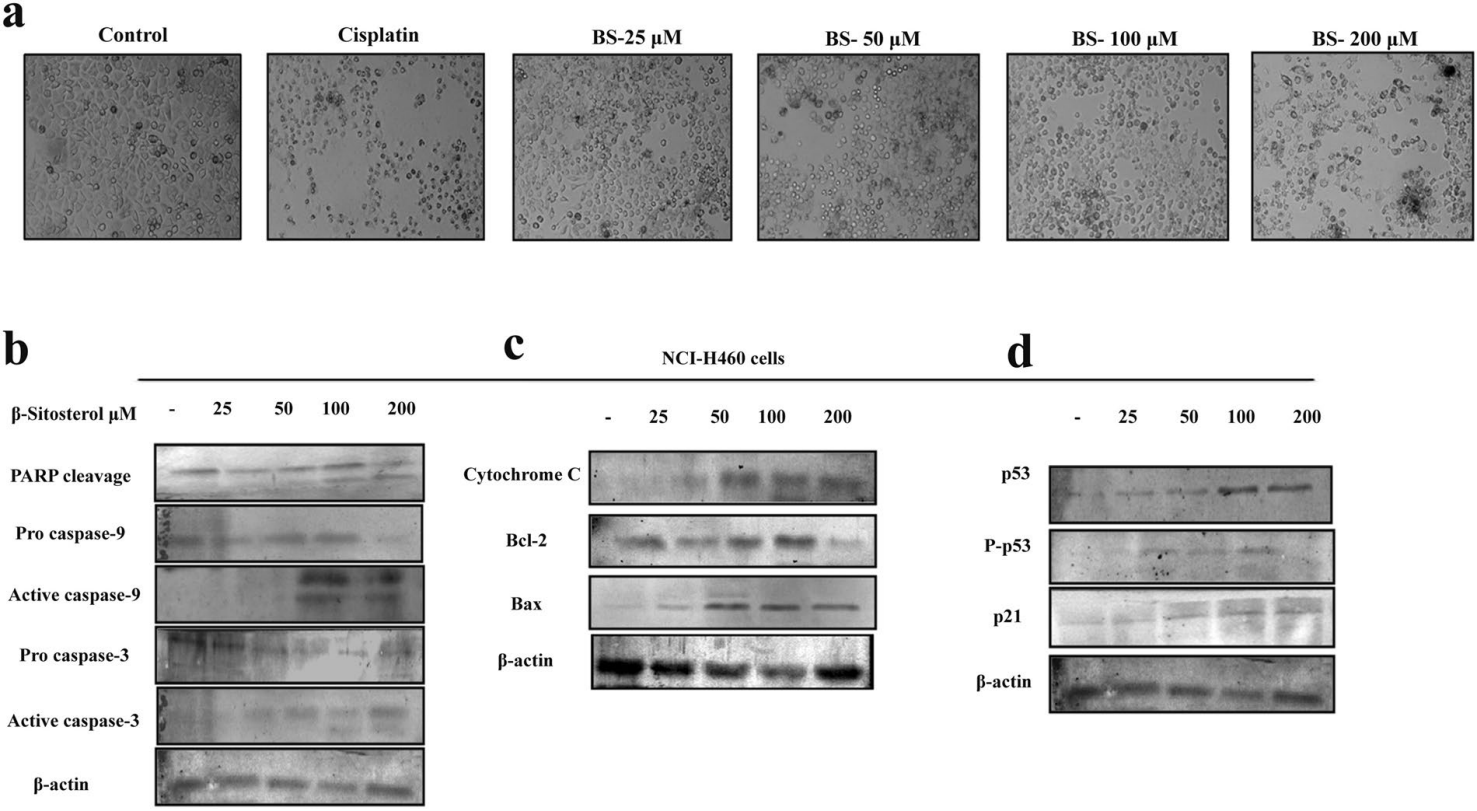
BS induces the intrinsic apoptotic pathway in NCI-H460 cells. (**a**) Light microscopic images of NCI-H460 cells after 72 h treatment of BS. (**b**) Expression of active caspases and PARP cleavage under BS induced condition on NCI-H460 cells. Significant up-regulation of caspase-3, caspase-9 and PARP cleavage indicated activation of apoptotic pathway upon BS treatment. (**c**) The expression level of mitochondrial mediated apoptotic regulators after 72 h time of BS exposure (**d**) The protein levels of p53, P-p53, p21 were evaluated by western blot analysis. The gel blots were cropped from different gels and the full length blots are given in the supplementary file.

To determine whether BS induced apoptosis was p53 dependent or independent, we knocked down the expression of p53 by pifithrin-α, that reversibly inhibit the p53 mediated apoptosis. A549 cells were co-incubated with BS and pifithrin-α for 72 h and the expression of p53, pSer15-p53 and p21was analyzed by western blot analysis. As shown in Fig. 7b, presence of pifithrin-α reversed the BS induced activation of p53, pSer15-p53 and p21 proteins. The down-regulation of p53 pathway also significantly affected the BS induced apoptosis in A549 cells which was evidenced by the absence of caspase 3, 9 activation and PARP inactivation. In addition, MTT experiments also evidenced that (Fig. 6c) the presence of pifithrin-α slightly affects the anti-proliferative efficacy of BS. Taken together, these results indicate that activation of p53 is essential for BS induced apoptosis in A549 cells. Moreover, we also verified the essential role of p53 through determining the cytotoxic effect BS in NCI-H460 (p53 wild) and NCI-H23 (p53 mutant) cells by MTT experiment. The results showed that (Fig. 7c & d), similar to A549 cells, the viability of NCI-H460 cells was significantly affected upon BS treatment, whereas the viability of NCI-H23 cells was not affected by BS exposure even after 72 h. To investigate whether the observation of p53 mediated apoptotic cell death is specific for A549 cells, we further examined apoptotic effect of BS on NCI-H460 cells. Intriguingly, our data suggested that similar observations (Fig. 8) were induced in BS treated NCI-H460 cells after 72 h time point. For instance, after BS treatment on NCI-H460 cells, cell shrinkage, elongation and reduced cell populations were observed (Fig. 8a). Meanwhile, significant elevation of active caspases 3 & 9 was found in BS treated NCI-H460 cells (Fig. 8b). Indeed, inactivation of PARP protein expression was also found in BS treated NCI-H460 cells. Together, the results clearly confirmed that BS induced intrinsic mediated apoptotic cell death in NCI-H460 cells also. To further substantiate the intrinsic apoptosis in NCI-H460 cells, we studied the expression of cytochrome c, bcl-2 and bax after 72 h of BS exposure. The results clearly showed (Fig. 8c) a reduction in the expression of bcl-2 protein and significant elevation of bax and cytochrome c, which confirmed that BS induced the intrinsic or mitochondrial mediated apoptotic pathway in NCI-H460 cells. Most importantly, the strong up-regulation of p53, pSer15-p53 and p21expression was observed in NCI-H460 cells (Fig. 8d) upon BS exposure at 72 h time point. Collectively, the results indicated that the activation of p53 is an essential step for BS mediated apoptosis in NSCLC cells.

### BS disturbs the redox status in A549 cells by targeting Trx/TrxR1

**Figure 9:**
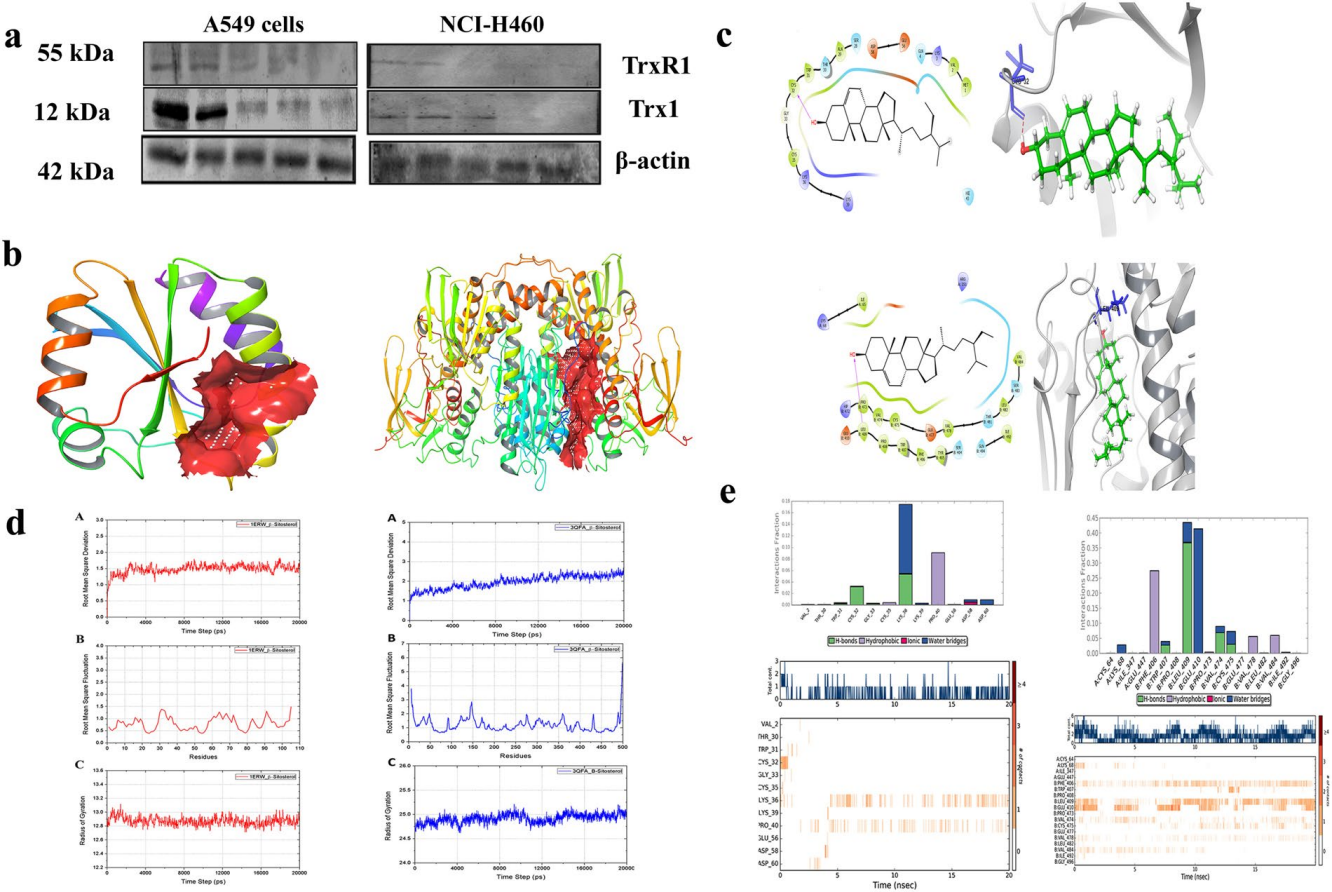
BS targets Trx/Trx1 reductase to generate ROS and triggers the apoptosis on A549 and NCI-H460 cells. (**a**) Protein expression of Trx and Trx1 reductase upon BS treatment with the indicated concentrations. (**b**) Result of Sitemap prediction on Trx/Trx reductase. Red surface shows the predicted binding site. (**c**) 2D and 3D interaction of Trx/Trx reductase docked with BS using Glide XP method. (**e**) RMSD, RMSF and Radius of gyration of Trx/Trx reductase complexed with BS over 20000 ps time period of simulation. (**f**) Histogram and Time line of Protein ligand contact of Trx/Trx reductase complexed with BS over 20000 ps time period of simulation. The gel blots were cropped from different gels and the full length blots are given in the supplementary file.

Hyper-activation of Thioredoxin (Trx1) and thioredoxin reductase **(**TrxR1) has been reported in numerous cancer cells to regulate their ROS homeostasis, promotes cell growth and induce apoptotic resistance^25^. Trx/Trx1 reductase has emerged as a novel drug target to selectively modulate the oxidative stress in cancer cells^26^. From the above experimental results, it is clear that ROS accumulation plays a crucial role in BS mediated mitochondrial dysregulation and p53 mediated apoptotic cell death. Therefore, we further investigated the role of TrxR1 and Trx1 by employing western blotting. It was interesting to note that, decreased expression of both TrxR1 and Trx1 (Fig. 9a) was found during BS treatment in A549 and NCI-H460 cells, which substantiate the possible deregulation of ROS homeostasis. The results were further confirmed through molecular docking and molecular dynamics studies which were performed to investigate the possible interaction of BS against the proteins Txr1 and TxrR1.

**Figure 10:**
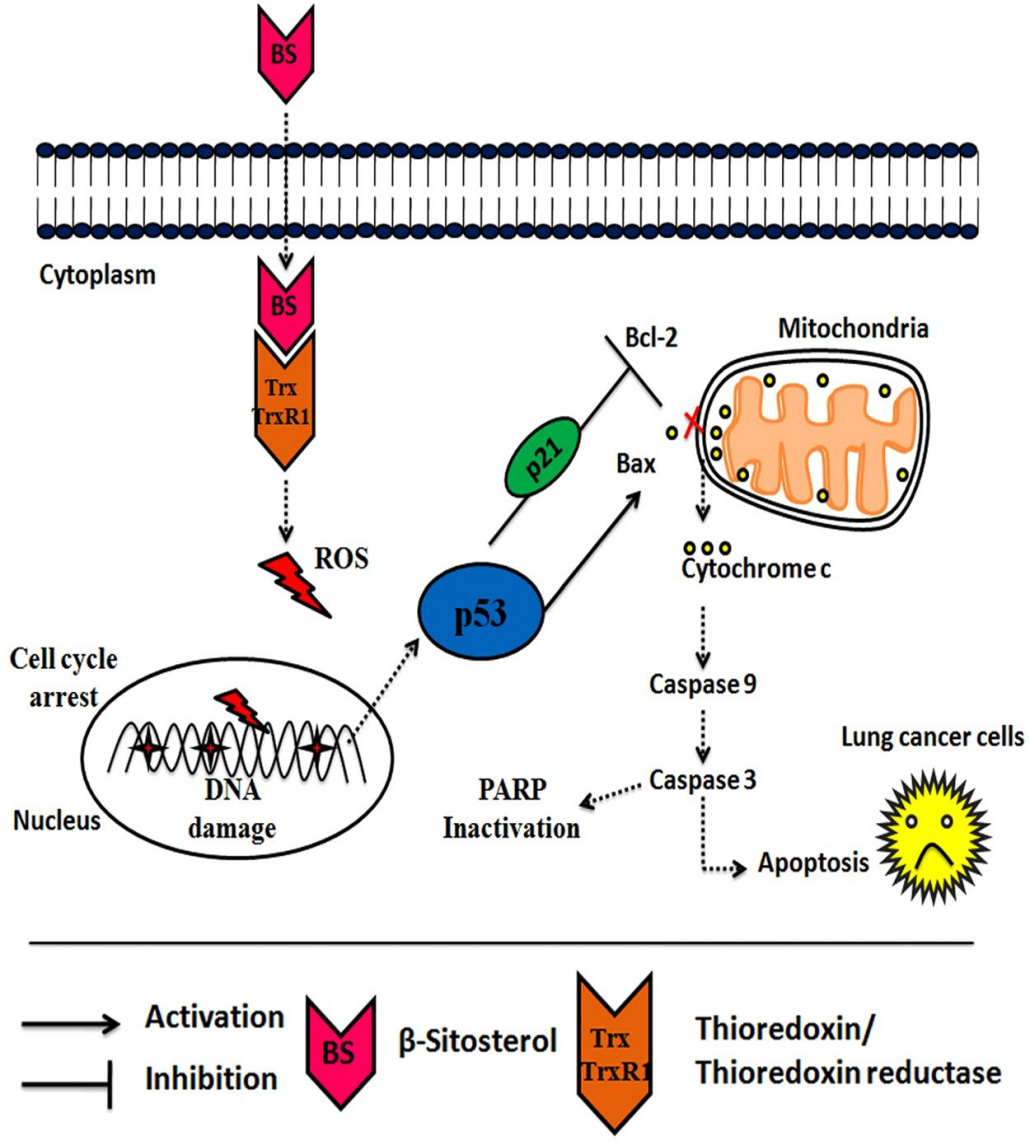
Schematic representation of the proposed signaling target of BS on NSCLC cells. BS induces excessive ROS production through inhibition of Trx/TrxR1. ROS accumulation causes DNA damage, p53/p21 activation and triggers the mitochondrial mediated apoptotic cell death in A549 and NCI-H460 cells.

Molecular docking analysis showed that BS interacts with Cys32 and Leu409 residues in Txr1 and TxrR1 respectively through hydrogen bond interaction (Fig. 9b & c). The docked complexes were further analyzed for molecular dynamics simulation for 20000 ps time period of simulation in order to access the binding interaction stability. The change in the structural integrity was analyzed through calculating the root mean fluctuations (RMSF) over backbone atoms. As shown in Fig. 9c, the BS-Txr1 complex is stable around 1.5 Å throughout the simulation which is well supported by RMSF in Fig. 9d. RGYR in Fig. 9d shows that the complex does not lose its compactness throughout the entire simulation. In the case of TxrR1-BS complex, the RMSD attained stability after 15000 ps around 2.5 Å. RMSF graph in Fig. 9c supported the RMSD result showing the average residue fluctuation of the protein. However, the protein does not lose its compactness, as the graph exhibited stable reading around 25 Å. Protein ligand contacts of Txr1 - BS complex clearly revealed (Fig. 9e) that Cys32 loses its interaction after few ps whereas Lys36 and Pro40 were predominant in the interaction with BS. Protein ligand contacts of TrxR1- BS complex clearly showed that Phe406, Leu409, Glu410 were predominant in interaction with the BS. Overall, the molecular dynamics study confirmed the strong and stable interaction of BS on Trx/Trx1 reductase proteins. Together, the present study concluded that BS induced the ROS dependent apoptotic mode of cell death in NSCLC cells through down-regulation of Trx/TrxR1 signaling pathway (Fig. 10).

## Discussion

Mortality and new cases of NSCLC is increasing rapidly in both developed and under developed countries. However, the crucial factor which contributes for the current therapy failure includes the series of side effects and the emergence of multi-drug resistance to the conventional chemotherapy drugs^27–29^. Therefore new therapeutic agents from the natural sources, with less toxic effects are currently being explored intensively, due to their versatile chemical availabilities. Our previous study demonstrated that, the Indian medicinal plant *Grewia tiliaefolia* leaf significantly affected the growth of NSCLC model A549 cell line by activating the intrinsic apoptotic pathway through the loss of ΔΨ m. In addition, β-sitosterol was identified as the major principle compound responsible for the anticancer action of the plant^30^. It has been reported that BS is effective against multiple kinds of cancers including breast, leukemia and prostate cancers^31^. However, BS had different targets among the different types of cancers to inhibit their growth. For instance, BS triggered apoptosis in leukemic cancer cells by affecting the microtubule dynamics through Bcl2-PI3K/Akt signaling pathway^11^. However, the apoptotic effect of BS on NSCLC cancer model is still not explored. In our present study, we have demonstrated that BS possesses anticancer activity in NSCLC models. Mechanistically, our data showed that BS induced apoptosis was associated with excessive ROS accumulation and down-regulation of Trx/TrxR1 redox system in A549 and NCI-H460 cells. To our knowledge, this is the first report demonstrating that BS induced apoptosis was associated with disturbance in redox status of cancer cells which gives new lead for further drug development.

Cell suicidal therapy (which induces apoptosis) is the most promising anti-cancer strategies in the clinical scenario, since it is effective against many types of human cancer. It increases the survival rate of the patients and delivers higher therapeutical outcome than the other treatments^32^. Apoptosis is a sequential genetic program that is involved in a diverse range of cellular process including tumor suppression. During the process of carcinogenesis, cancer cells modulate several key proteins and develops resistance against apoptotic death^33^. Hence, developing anticancer agents that promotes apoptotic cell death in tumor cells has been intensively investigated in the last few decades by scientific and medical communities^34^. In this study, we found severe cell membrane damage, signature morphological changes and cell cycle arrest at sub-G1 phase in A549 cells, which positively indicated that BS induced growth inhibition occurred via activation apoptotic cell death. Indeed, we also investigated on whether apoptosis or necrosis is involved in the anti-cancer mechanism of BS by Annexin-V/FITC and PI staining. Translocation of phosphatidylserine to the extracellular surface of cell membrane is wildly recognized as the signaling marker for cells undergoing apoptotic mode of cell death. Annexin-V/FITC and PI staining confirmed the formation of increased percentage of early apoptotic cells during treatment with the minimum dose of BS. On the other hand, a dose dependent increase of late apoptotic cells were observed without the formation of any necrotic cells.

Caspases, which are the family of cysteine-aspartic proteases plays a crucial role in executing the apoptosis mechanism^35^. Activation of caspases leads to cell membrane damage and formation of apoptotic bodies. The results of SEM images and morphological changes confirm the severe cell membrane damage induced upon BS treatment, which may have occurred via active caspases. Hence the expression of active caspases 3 & 9 were determined upon BS treatment and the results showed a significant up-regulation of active caspase-3 in A549 cells. Indeed, activation of caspase-3 was also consistent with inactivation of PARP protein, which has been demonstrated as an ideal biological marker in many chemotherapeutical drugs exposure and embryonic development. PARP (Poly (ADP-ribose) polymerase) is a family of protein, which gets activated during DNA repair, apoptosis, transcription and recombination^36^. The cleavage of PARP protein during BS treatment in A549 cells confirmed the activation of caspases by BS. It is well established that both intrinsic and extrinsic apoptotic pathways are executed by different caspases. Caspase-9 is involved in the initiation and execution of intrinsic apoptosis pathway^37^. The up-regulation of active caspase-9 was observed in A549 cells which positively indicated that BS induced apoptosis was mediated through intrinsic apoptotic pathway.

To further confirm the activation of intrinsic apoptotic pathway by BS, we studied mitochondrial specific apoptotic regulators. Bcl-2 family of proteins are the key players governing both intrinsic and extrinsic apoptotic pathway through maintaining mitochondrial permeability^38^. Bcl-2 family consists of both pro –apoptotic and anti-apoptotic proteins which controls the release of apoptotic factors (Cytochrome c) into the cytosol. Disruptions in the ΔΨ m by external agents like ROS or chemotherapeutic drugs leads to release of the apoptogenic factors in the cytoplasm, which in turn activates the intrinsic apoptotic pathway^39,40^. In the present study, loss in ΔΨ m was observed in A549 cells upon BS treatment even at 6 h, and it peaked at 48 h, which substantiates that loss of ΔΨ m is an earlier event in BS induced apoptosis. Concomitantly, BS treatment increased the expression level of pro-apoptotic protein bax and significantly decreased the expression of anti-apoptotic protein bcl-2. Constitutive expression of bcl-2 was observed in numerous primary tumors, which play an important role in tumor cell survival, growth and apoptotic resistance. However, our results showed complete down-regulation of bcl-2 protein and dose dependent increase of bax protein. These results suggested that BS induced apoptosis in A549 cells by promoting MMP loss and alteration in bcl-2-bax ratio. Cytochrome c is a large transmembrane protein which generally resides in the mitochondrial membrane space and released into the cytosol upon apoptosis activation or MMP loss which further triggers intrinsic mode of apoptosis. Interestingly, the release of cytochrome c following BS exposure witnessed the loss of mitochondrial transition pore and activation of intrinsic mode of apoptotic mode of cell death in A549 cells. Collectively, the results confirmed that the loss of ΔΨ m corresponds with the onset of early events in BS induced apoptosis.

To assess whether the loss of ΔΨ m was associated with generation of ROS, the ROS level was determined at different time points upon BS treatment. Accumulation of ROS was observed at 6 h of BS treatment and it peaked after 48 h. However on addition of the ROS scavenger (NAC), the cytotoxic effect of BS was significantly affected in the A549 cells. It is well established that mitochondria are the major intracellular organelle that generates ROS upon mitochondrial respiration in mammalian cells^41^. Indeed, cancer cells are characterized by increased aerobic glycolysis (Warburg effect) and makes oxidative stress environment through excessive ROS production^42^. However, cancer cells combat the higher ROS levels through improved antioxidant defense mechanism and aberrant signaling pathways. Thus, modulating redox status in tumor cells may offer for selective elimination of cancer cells while sparing normal counterpart^20^. Accumulation of ROS and ΔΨ m loss in A549 cells were observed during the initial hours after BS treatment, which suggested that BS could affect the redox status in A549 cells and elevate the ROS level. Taken together, the results strongly supported that the loss of ΔΨ m was associated with increased ROS production during BS treatment.

ROS is a highly reactive molecule that is involved in cell proliferation and differentiation, whereas excessive ROS is detrimental to the cells and promotes apoptotic cell death by modifying the cellular macromolecules like DNA, protein and lipids^43^. Comet assay and DAPI staining revealed DNA strand breaks in A549 cells, which confirmed the excessive accumulation of ROS upon BS treatment. Moreover, abnormal ROS also damaged mitochondrial membrane permeability. Meanwhile, the presence of NAC significantly eliminated ROS induced DNA damage and cytotoxic effect of BS in A549 cells. The results confirmed the excessive accumulation of ROS and DNA damage, which suggested that it could contribute for the mitochondrial permeability loss and release of apoptogenic factors during BS exposure in A549 cells.

Multiple lines of evidences support that DNA damage and excessive ROS release promotes apoptosis through regulating various signaling pathways^44^. Since, cell cycle arrest and DNA damage was noticed during BS treatment, it indicated the possible involvement of p53 pathway. P53 is a major tumor suppressor protein, the expression of which was found to be silenced in numerous cancer tissues and cell lines^45^. Hence reactivation of p53 expression is the most promising approach in anticancer chemotherapeutics^46^. Interestingly, our results showed that the expression of both p53, pSer15-p53 (phosphorylated p53) was activated, which indicated that ROS induced apoptosis has occurred via p53 regulation during BS treatment. In addition, activation of p21^Cip1^ was also observed upon BS treatment. p21^Cip1^ comes under the family of CKIs (Cyclin dependent kinase inhibitors) which is activated by p53 upon DNA damage^47^. Since p21 activation leads to cell cycle arrest at G1, G2 or S phase, the results suggest that BS induced ROS mediated DNA damage triggers the activation of p53 and arrest the cell cycle via p21 activation. To further investigate the role of p53, we studied the apoptotic effect of BS in the presence of p53 inhibitor. Western blot analysis clearly showed that the presence of p53 inhibitor significantly affected the BS induced activation of p53, pSer15-p53 (phosphorylated p53) and caspase-3, 9 cleavages. Concomitantly, p53 inhibitor also affected the cytotoxic effect of BS on A549 cells. Interestingly, BS showed less cytotoxic effect in p53 mutant lung cancer cell line NCI-H23, while increased cytotoxic effect was observed in p53 wild lung cancer cell line NCI-H460. Meanwhile, our data suggested that BS also induced the mitochondrial mediated apoptotic cell death in NCI-H460 cells by p53 activation and phosphorylation. Collectively, our results suggest the important role of p53 in regulating BS induced apoptosis on NSCLC cancers.

Regulation of redox homeostasis is a fundamental phenomenon for normal cellular functions and survival. Growing evidences suggested that the tumor cells have excessive ROS production than normal cells, due to its aberrant cell metabolism and continuous cell division. However, tumor cells have a well adapted redox homeostasis mechanism to survive in the oxidative stress conditions^48^. Mammalian thioredoxin reductase system (TrxR) plays a crucial role in regulating the multiple redox based signaling pathways and have attracted increased attention in designing the cancer drug targets^26,49–51^. Enhanced expression of TxrR1 has been observed in many primary tumors, suggesting that tumor cells are adapted to survive and proliferate^52,53^. Hence, we investigated the underlying mechanism of ROS accumulation by monitoring the protein expression of both Trx1 and TrxR1. It was observed that the expression of both Trx/TrxR1 was downregulated upon BS treatment in both A549 and NCI-H460 cells. Concomitantly, strong and stable interaction of BS on both Trx/TrxR1 was also observed in molecular docking and dynamics studies. On the whole, the results strongly supported that BS modulated the TrxR redox system to generate excessive ROS in A549 cells to promote apoptosis. Collectively, the present study disclosed that BS triggered apoptosis through ROS dependent pathway, which was mediated through Trx/TrxR1 signaling pathway.

## Materials and Methods

### Reagents and antibodies

(3-(4, 5-Dimethylthiazol-2-yl)-2, 5-diphenyl tetrazolium bromide (MTT), DMSO, Lactic acid, NAD^+^, Gluteraldehyde, NAC were purchased from Himedia (India). Propidium iodide, DCF-DA, Rhodamine 123, Pifithrin-α, Anti-rabbit IgG secondary antibody were purchased from Sigma (St. Louis, MO, USA). β-Sitosterol was purchased from Cayman Chemicals (Michigan, USA). Apoptosis kit (V13242) was purchased from Thermo Fisher Scientific, USA. Antibodies against β-actin, caspase-3, caspase-9, Bcl-2, Bax, p21, p53, P-p53, cyclin d, cdk-6, Trx were purchased from Santa Cruz Biotechnology Inc. (Dallas, TX, USA). Antibodies against PARP, Cytochrome-c, TrxR were purchased from Cell Signaling Technology (Danvers, MA, USA).

### Cell lines and cell culture

A549, L132, NCI-H460, NCI-H26 were purchased from National Center for Cell Science, Pune, India. A549 and L132 cells were cultured in Ham’s F12 and DMEM medium respectively (GIBCO, Invitrogen). NCI-H460 and NCI-H26 cells were cultured in RPMI-1640 (Himedia, India). The medium contained 10% FBS (GIBCO, Invitrogen), 1× Antibioic-Antimycotic solution (GIBCO, Invitrogen). All the cells were maintained in humidified condition with 5% CO_2_ at 37 °C.

### MTT assay

Cell viability was assessed with MTT assay as previously described^30^. Briefly, A549 cells (5 × 10^4^) were seeded in 96 well plate (Nunc) for initial attachment and then incubated with different concentrations of BS at various time intervals. After incubation, medium was removed and cells were incubated with freshly prepared MTT (1 mg/ml) for 3 h. The formazan salt was dissolved with 100 μL of DMSO and the absorbance was taken at 570 nm using Multi Label Reader spec (Molecular Device Spectramax M3, equipped with Softmax Pro V5 5.4.1 software). The same protocol was followed to assess the BS cytotoxicity in L132, NCI-H460, NCI-H23 cells. Cytotoxicity of BS on PBMC cells was assessed by trypan blue dye exclusion method describes as earlier^30^.

#### Ethical statement for collection of human PBMC

Informed consent letter was obtained from the blood donors. The experimental protocol was approved by the Institutional Ethics Committee of Alagappa University, Karaikudi, India (Approval No. IEC/AU/2016/1/11) and the blood for PBMC isolation was collected in accordance with The Code of Ethics of the World Medical Association (Declaration of Helsinki).

### LDH assay

Leakage of LDH upon BS treatment was measured by LDH assay as described ealier^54^. After BS treatment, cell free supernatant was collected and incubated with 100 μL of reaction mixture to give a final concentration of 3.75 mM NAD^+^ and 25 mM L-lactic acid in 125 mM Tris-HCl buffer, pH 8.9 in 96 well plates. The absorbance was measured at 340 nm using Multi Label Reader spec (Molecular Device Spectramax M3, equipped with Softmax Pro V5 5.4.1 software). The percentage of LDH leakage was calculated by comparing the values with total LDH activity of untreated A549 cells lysed with 0.2% Triton X-100.

### Cell cycle analysis

Cell cycle distribution was evaluated using flow cytometry analysis^30^. Briefly, after BS exposure the A549 cells were trypsinized and washed with ice cold PBS for three times. The cell pellet was suspended in 70% ethanol (v/v) for O/N at −20 °C. Further, the fixed cells were washed with PBS and stained with PI (50 μg/ml), RNaseA (100 μg/ml) for 30 minutes in dark condition. Stained cells were then analysed by flow cytometry (BD FACSCalibur™).

### Apoptosis detection by Annexin-V/FITC and PI staining

Translocation of phosphatidylserine in BS treated cells was assessed through Annexin-V/FITC and PI staining according to the manufacturer protocol. After BS treatment, cells were washed with ice-cold PBS and suspended in 1× Annexin-V binding buffer. Further, cells were stained with 5 μL of FITC Annexin-V and 1 μL of the PI stain (100 μg/mL), and incubated in dark condition for 15 min. After the incubation time, apoptotic and necrotic cells were analyzed by fluorescence microscopy (Nikon ECLIPSE, Ti-E, Japan).

### Measurement of mitochondrial membrane potential (ΔΨ m)

Changes in the ΔΨ m upon BS treatment was analyzed using Rhodamine 123 staining as described earlier^30^. Briefly, A549 cells were treated with IC_50_ concentration of BS for different time points (0, 6, 12, 24 and 48 h). After the incubation time, cells were trypsinized and fixed in 4% paraformaldehyde for 10 min. The fixed cells were stained with 100 μL of Rhodamine 123 (10 μg/ml) for 10 minutes. After incubation, the dye was removed and cells were washed twice with ice-cold PBS. Fluorescence intensity was quantified using fluorescence spectroscopy (Molecular Device Spectramax M3) and supportive images were taken in fluorescence microscopy (Nikon ECLIPSE, Ti-E, Japan).

### Measurement of ROS generation

Generation of ROS upon BS treatment was analysed by DCF-DA method as previously described^55^. Briefly, A549 cells treated with IC_50_ concentration of BS for various time points (0, 6, 12, 24 and 48 h). After every incubation time, A549 cells were harvested and stained with 300 μL of DCF-DA (10 μg/ml) and incubated in dark for 10 minutes. After the incubation time, A549 cells were washed with ice-cold PBS and the fluorescence intensity was measured in fluorescence spectrophotometer with the respective images taken in fluorescence microscopy.

### Single cell gel electrophoresis

DNA damages in A549 cells under BS treatment was measured by comet assay as previously described^30^. In brief, A549 cells (1 × 10^7^) were incubated with different concentrations of BS for 72 h. After incubation, the cells were centrifuged at 1500 g for 10 min and the pellet was suspended in low melting agarose (Himedia). Further, it was poured into glass slides which were precoated with normal melting agarose. The agarose slides which were embedded with the cells were incubated for 10 min at 4 °C and immediately placed in lysis buffer (10% SLS, 5 M NaCl, 0.5 M EDTA, Tris-pH 10, 1% triton-X, 10% DMSO) for overnight. The slides were then electrophoresed with freshly prepared pre-cooled buffer for 20 min at 25 V which was further soaked in neutralization buffer. All the slides were stained with ethidium bromide and the images were taken in confocal laser scanning microscopy (Model: LSM 710, Carl Zeiss, Germany). The comet score was measured using Open Comet Score (Open Comet v1.3.1) and the comet parameters like tail moment, olive moment and percentage of DNA in tail were calculated.

### Western blot

Cells were trypsinized and lysed in NP40 lysis buffer in the presence of protease inhibitor cocktail. Protein concentration was measured by Bradford method and proteins (50 μg) were separated on 12% SDS-PAGE gel. Separated proteins were blotted on PVDF membrane and blocked with 5% BSA for O/N. Further, the membrane was incubated with respective primary antibodies for 6 h and detected with alkaline phosphatase conjugated secondary antibody. Detected bands were captured in gel doc system and quantified using ImageJ software.

### Molecular docking and dynamics

*In silico* analysis was carried out on a High Performance Workstation operated with Cent OS Version-6.5 Linux operating platform. Hardware specifications are HPC workstation running with Intel core i7 processor of 8 Cores and 16 GB RAM speed. Software specifications used are commercial version of Schrödinger software package, LLC, New York, NY, 2017. Detailed methodology is given in Supplementary File.

### Statistical analysis

The statistical comparison was done using SPSS statistical package (SPSS 17.0 for Windows; SPSS, Inc. USA) and difference between two groups were analysed by two-tailed Student’s t-test. All the experimental results were presented as mean ± S.D^56–63^.

## Electronic supplementary material

Supplementary Information
