# Supplementary material for: β-Sitosterol targets Trx/Trx1 reductase to induce apoptosis in A549 cells via ROS mediated mitochondrial dysregulation and p53 activation
Source: Sci Rep. 2018 Feb 1;8:2071. doi: 10.1038/s41598-018-20311-6 (PMC5794769; doi:10.1038/s41598-018-20311-6)
Supplement: Supplementary file 1 — Supplementary Information [file 41598_2018_20311_MOESM1_ESM.docx]

**Supplementary Information**

β-Sitosterol targets Trx/Trx1 reductase to induce apoptosis in A549 cells via ROS mediated mitochondrial dysregulation and p53 activation

Tamilselvam Rajavel^1^, Pandian Packiyaraj^1^, Venkatesan Suryanarayanan^2,^ Sanjeev Kumar Singh^2,^ Kandasamy Ruckmani^3^, Kasi Pandima Devi^1*^

^1^Department of Biotechnology, Science Campus, Alagappa University, Karaikudi-630 003, Tamil Nadu, India.

^2^ Computer Aided Drug Design and Molecular Modeling Lab, Department of Bioinformatics,

Alagappa University, Karaikudi- 630003, Tamil Nadu, India

^3^ National Facility for Drug Development (NFDD) for Academia, Pharmaceutical and Allied Industries, Bharathidasan, Institute of Technology, Anna University, Tiruchirappalli, 620024, Tamil Nadu, India.

^*^ Corresponding author: Kasi Pandima Devi; E mail: devikasi@yahoo.com

**Materials and Methods**

**System Configuration**

All the research works were carried out on a High Performance Workstation operated with Cent OS Version-6.5 Linux operating platform. Hardware specifications are HPC workstation running with Intel core i7 processor of 8 Cores and 16 GB RAM speed. Software specifications used are commercial version of Schrödinger software package, LLC, New York, NY, 2017.

**Protein and ligand preparation:**

The crystal structure of target protein Thioredoxin and Thioredoxin reductase, (PDB ID: 1ERW and 3QFA respectively) was retrieved from Protein Data Bank (PDB). The typical structure file from the PDB is not suitable for immediate use in molecular modeling calculations, so both the crystal structures were prepared through Protein Preparation Wizard, implemented in Maestro 11.3. Missing residues were added using the Prime loop modeling, which is also embedded in Protein Preparation Wizard. First, the bond orders were assigned, hydrogen atoms were added, and all the crystallographic waters molecules were removed. The protassign script optimizes the hydrogen-bonding network, rotating hydroxyl and thiol hydrogens, generating appropriate protonation and tautomerization states of HIS, and performing chi flips in ASN, GLN, and HIS residues. Optimized structure of ACHE was minimized using OPLS-2005 force field, until the average root mean-square deviation (RMSD) of the non-hydrogen atoms reached 0.3Å. 2D structure of β-Sitosterol was retrieved from Pubchem. It was optimized and converted into 3D structure using Ligprep.

**Binding Site Prediction**

The binding site of the target protein was investigated using the SiteMap program which generates binding site’s characteristic information using novel search and analytical facilities. SiteMap calculation begins with an initial search step that characterizes one or more regions on the protein surface that may be suitable for binding ligands to the receptor. Contour maps are then generated, producing hydrophobic and hydrophilic maps. The hydrophilic maps are further divided into donor, acceptor and metal-binding regions. The evaluation stage, which concludes the calculation, involves assessing each site by calculating various properties: the number of site points, a measure of the size of the site; exposure/enclosure, two properties providing different measures of how available the site is to the solvent; contact, which measure how strongly the average the site point interacts with the surrounding receptor via van der Waals nonbonding interaction; donor/acceptor character, a property related to the sizes and intensities of H-donor and H-acceptor regions and Site Score an overall property based on the previous properties, constructed and calibrated so that the average Site Score for a promising binding site is 1.0

**Molecular docking**

The docking protocol was carried out using Glide. After ensuring that the protein and ligands were in the correct form for docking, the receptor-grid files were generated using a grid-receptor generation program. In this study, grid was generated by using the predicted active site through sitemap. Ligand docking panel was used to carry out the docking of prepared ligands. The receptor grid generated file was uploaded and the precision XP was selected. Glide score was generated based on the following formula.

GScore=a*vdW + b* Coul + Lipo + Hbond + Metal +BuryP + RotB + Site. (1)

Where, vdW=van der Waal energy, Coul=Coulomb energy, Lipo=lipophilic contact term, Hbond=hydrogen-bonding term, Metal=metal-binding term, BuryP=penalty for buried polar groups, RotB= penalty for freezing rotatable bonds, Site=polar interactions at the active site^56^.

**Binding Free Energy Calculation**

The free energy of binding was calculated using Prime MM-GB/SA approach. In this approach, the docked poses were minimized using the local optimization feature in Prime and the energies of complex were calculated using the OPLS-AA (2005) force field and generalized-Born/surface area (GB/SA) continuum solvent model. The free energy of binding, ΔG_bind_ is calculated as^57,58,59^

ΔG_bind_ = ΔE + ΔG_solv_ + ΔG_SA_ (2)

ΔE= E_complex_– E_protein_– E_ligand_ (3)

where, E_complex,_ Eprotein_,_ and E_ligand_ are the minimized energies of the protein-ligand complex, protein, and ligand, respectively. Prime uses a surface generalized Born (SGB) model employing a Gaussian surface instead of a van der Waals surface for better representation of the solvent-accessible surface area.

ΔG_solv_= G_solv_(complex) - G_solv_(protein) - G_solv_(ligand) (4)

where, G_solv_(complex), G_solv_(protein), and G_solv_(ligand) are the solvation free energies of the complex, protein, and ligand, respectively.

ΔG_SA_ = G_SA_(complex) - G_SA_(protein) - G_SA_(ligand) (5)

where, G_SA_(complex), G_SA_(protein), and G_SA_(ligand) are the surface area energies for the complex, protein, and ligand, respectively. The rational criteria for selection of best compounds based on scoring and interaction parameters shown in XP docking with different charge model of ligands.

**Molecular Dynamics Simulation**

The structure of Trx1 and TrxR1 was retrieved from Protein Data Bank. In order to procure the interaction stability of the docked complex, Molecular Dynamics simulation was performed for both thioredoxin and thioredoxin reductase with β-Sitosterol using Desmond with Optimized Potentials for Liquid Simulations (OPLS) 2005 force field^60^. The systems were imported in Desmond system setup wizard and solvated in an orthorhombic periodic box of TIP3P water molecules and neutralized using appropriate number of counter ions and 0.15M of salt concentration^61,62,63.^ A distance of 10 Ǻ was set between the box wall and protein complex to avoid direct interaction with its own periodic image. Steepest descent method was used to minimize the energy of prepared systems with a maximum of 5000 steps until a gradient threshold (25kcal/mol/Ǻ) is reached. The systems were equilibrated using the default protocol provided in Desmond. The equilibrated systems were further carried to perform MD simulations for 100 ns (100000 ps) at constant temperature of 300 K and constant pressure of 1 atm with a time step of 2 fs.

**Figure S1. The full length blots or original images for Figure 2c**

**BS (μM)**

**C 25 50 100 200**


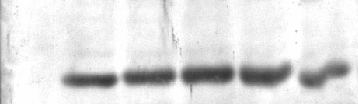


**β-Actin**


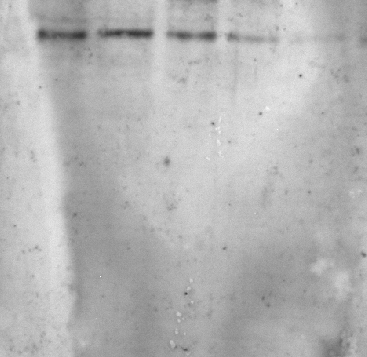


**CDK-2**

**Non-specific bands**

**Cyclin D1**


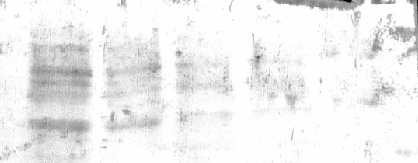


**Figure S2. The full length blots or original images for Figure 3c & 4c**

**BS (μM)**

**C 25 50 100 200**


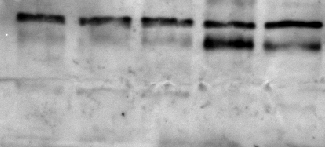


**PARP**


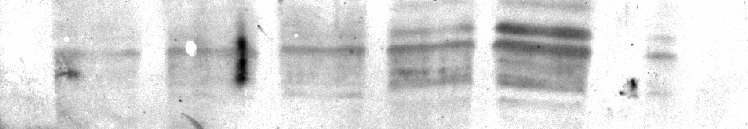


**Active Caspase-3**


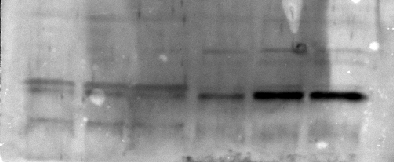


**Active Caspase-9**


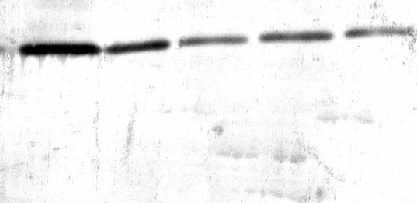


**Bcl-2**


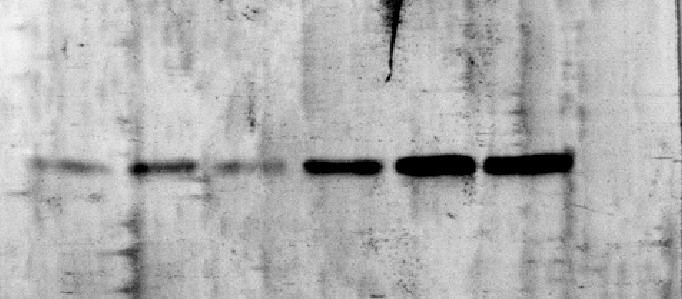


**Bax**


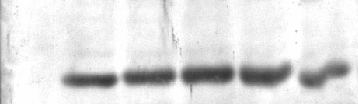


**β-Actin**

**Figure S3. The full length blots or original images for Figure 7a**

**BS (μM)**

**C 25 50 100 200**


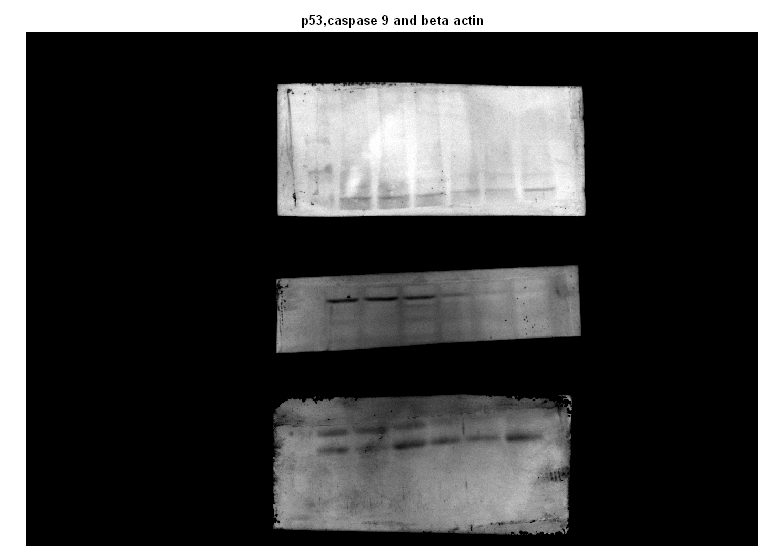


**P53**


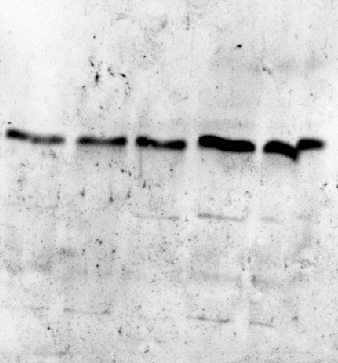


**P-p53**


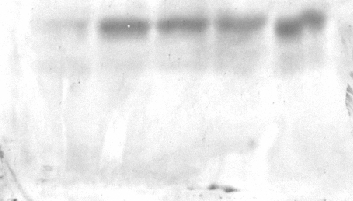


**P21**


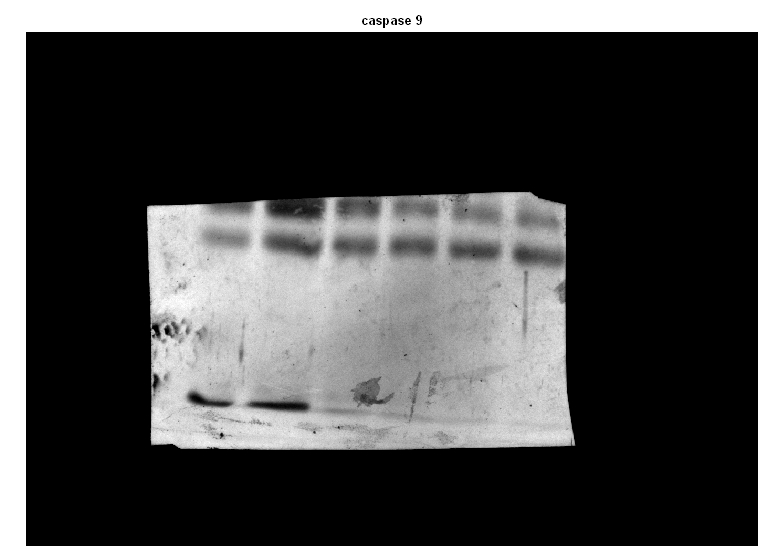


**β-Actin**

**Figure S4. The full length blots or original images for Figure 7b**

**BS**

**- + + -**

**Pifithrin-α**

**- - + +**


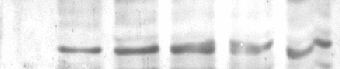


**P53**


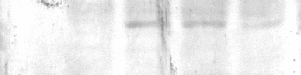


**P-p53**


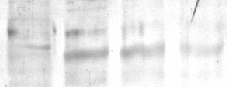


**P21**


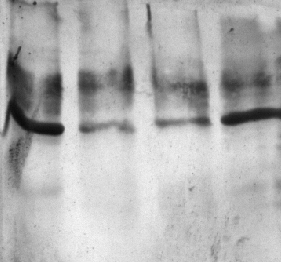


**PARP**


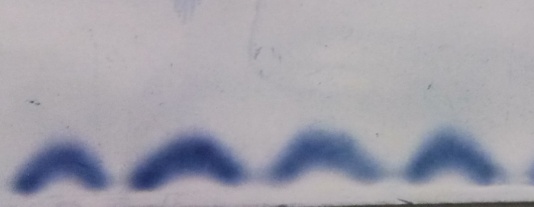


**Active caspase-9**


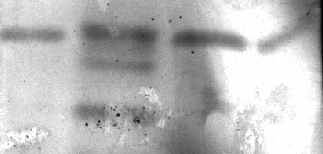


**Active caspase-3**


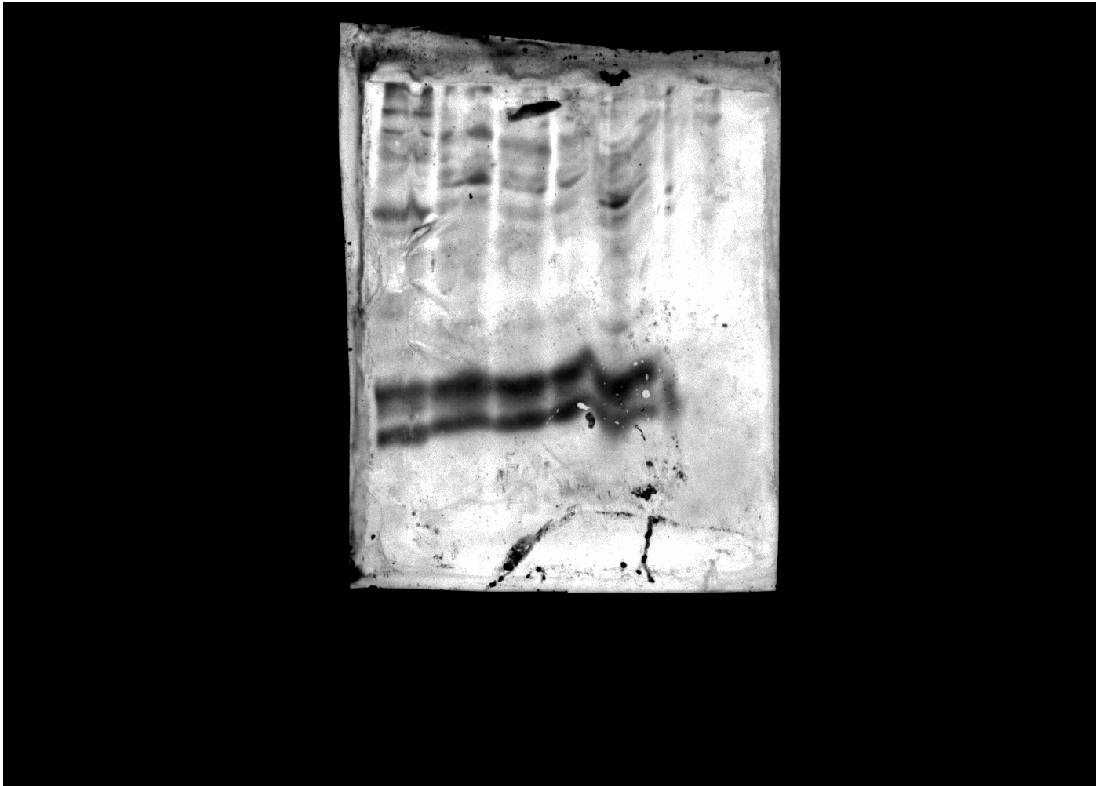


**β-Actin**

**Figure S5. The full length blots or original images for Figure 9a**

**C 25 50 100 200**

**BS (μM)**

**
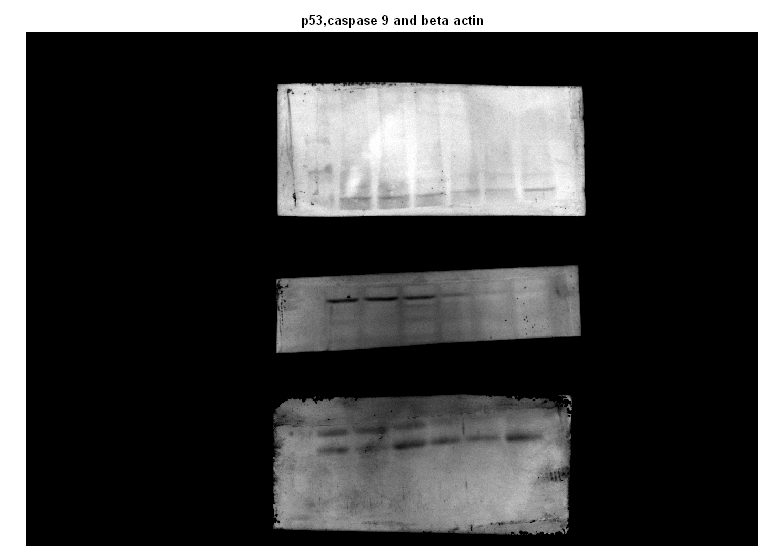
**

**PARP Cleavage**

**
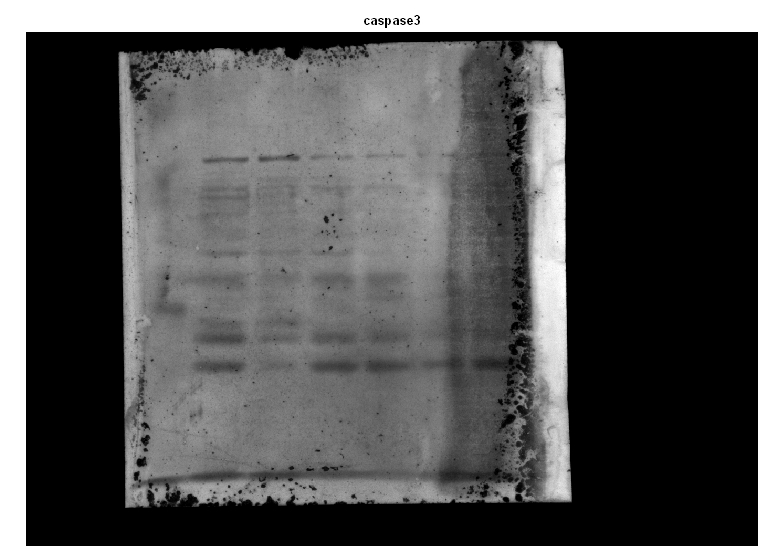
**

**Pro Caspase-9**

**
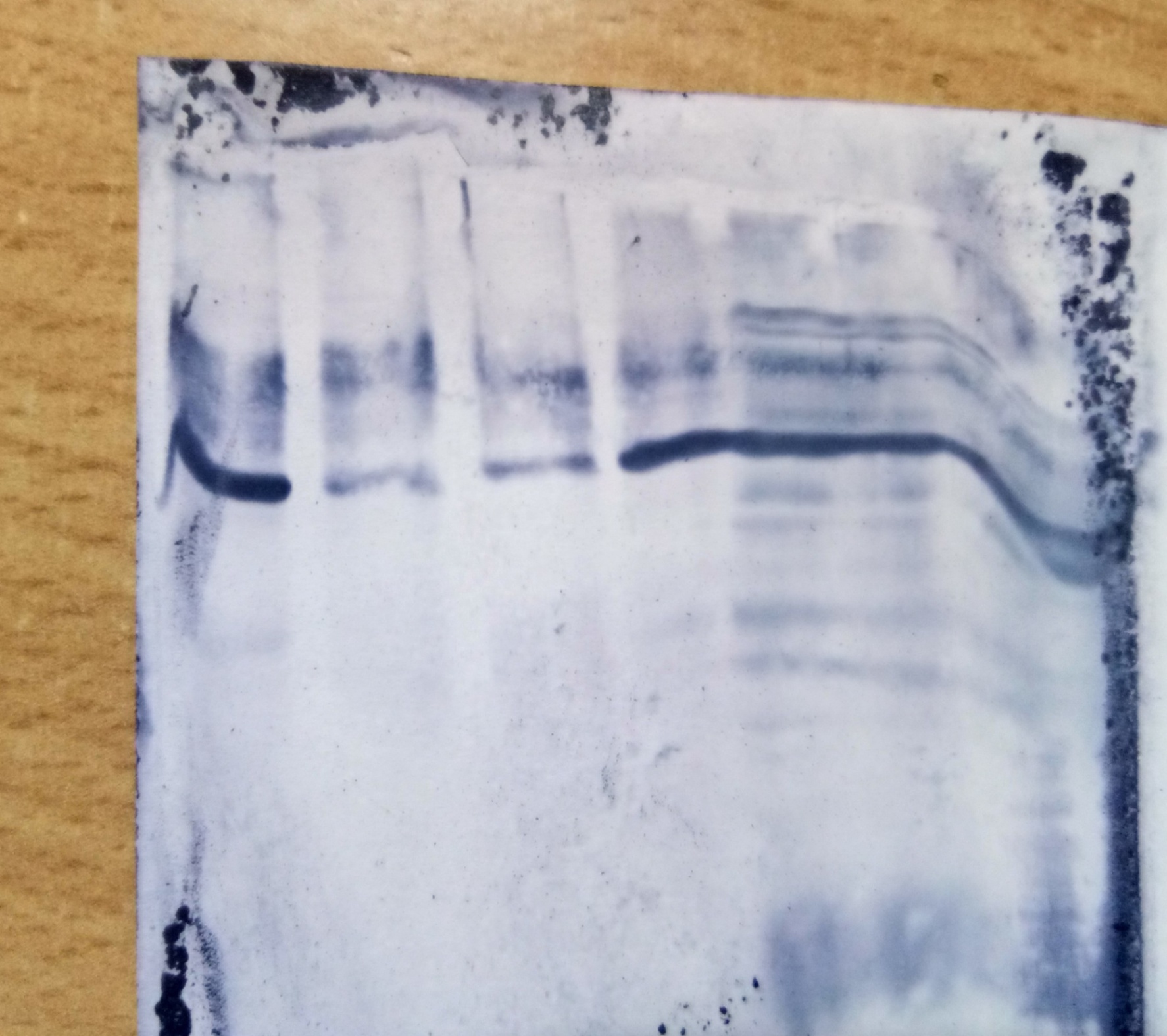
**

**Active Caspase-9**

**
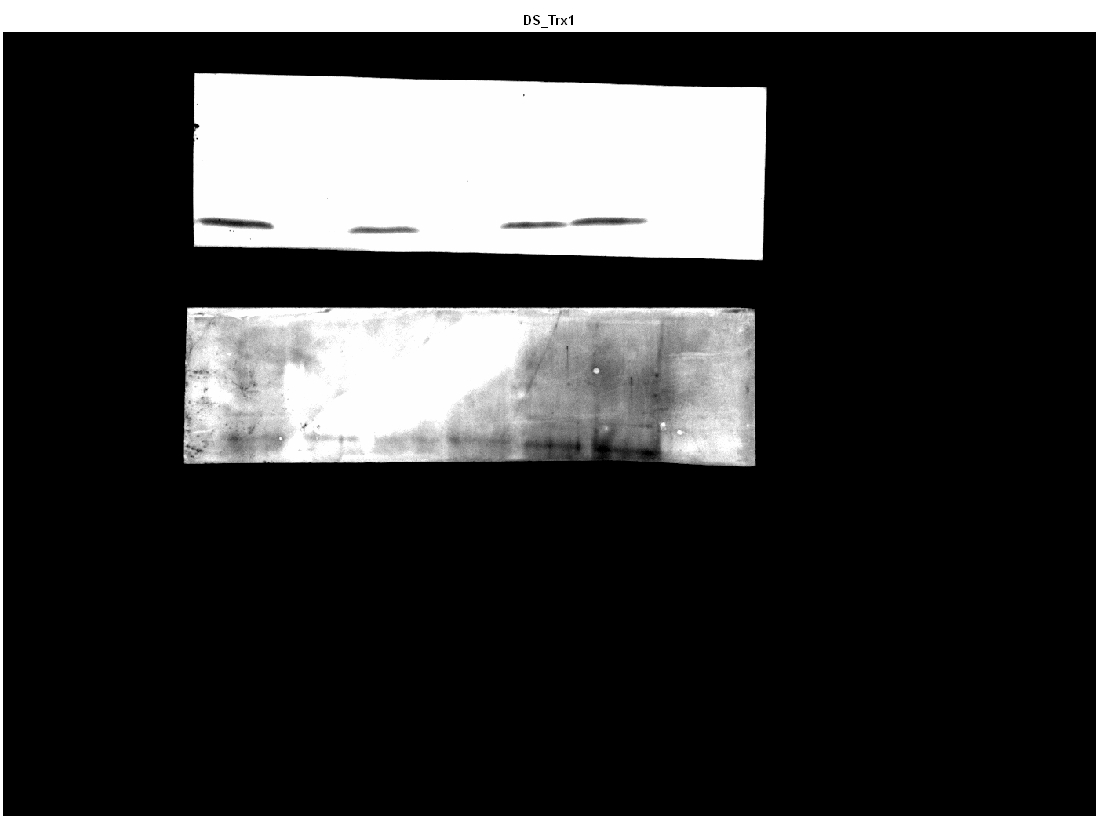
**

**Pro Caspase-3**

**
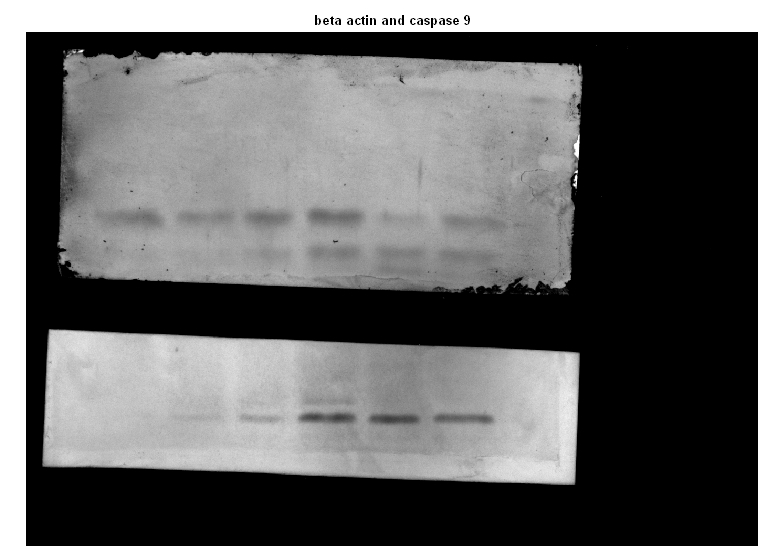
**

**Active Caspase-3**

**
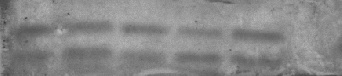
**

**β-actin**

**
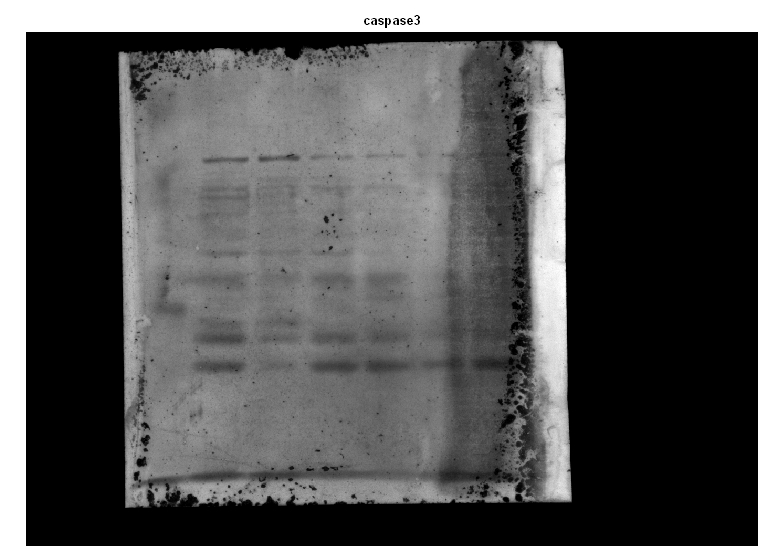
**

**Bcl-2**

**
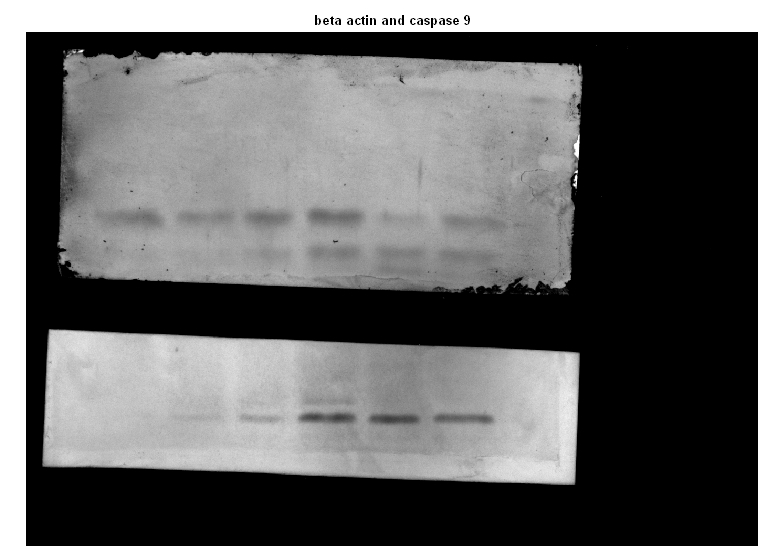
**

**Bax**

**
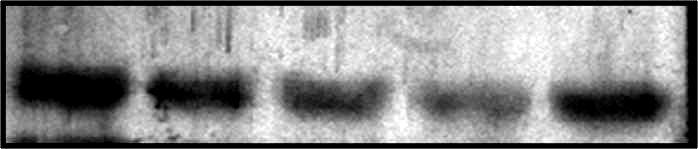
**

**β-actin**

**
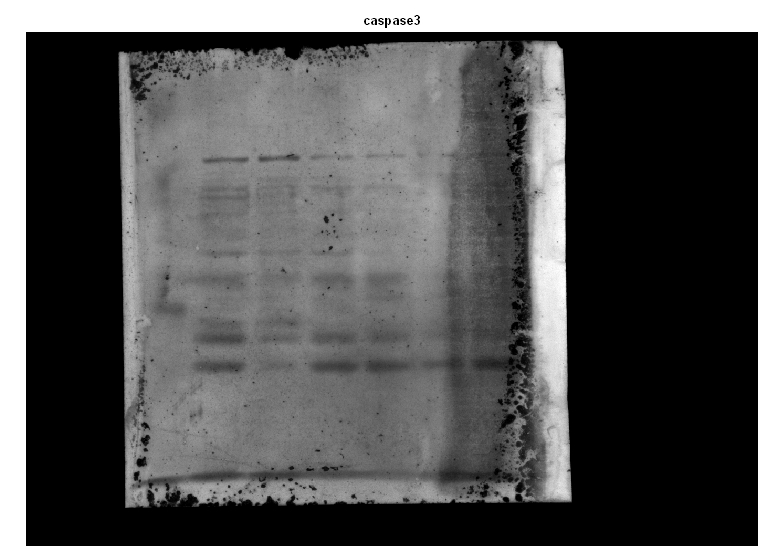
**

**p53**

**
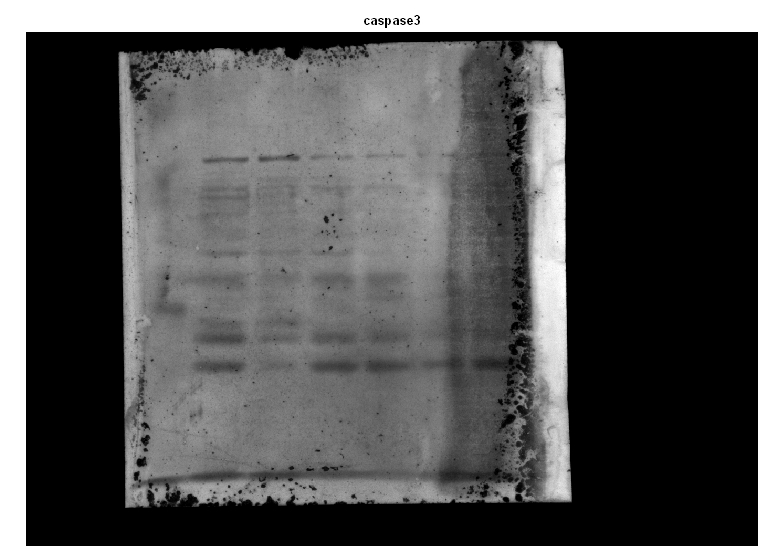
**

**P-p53**

**
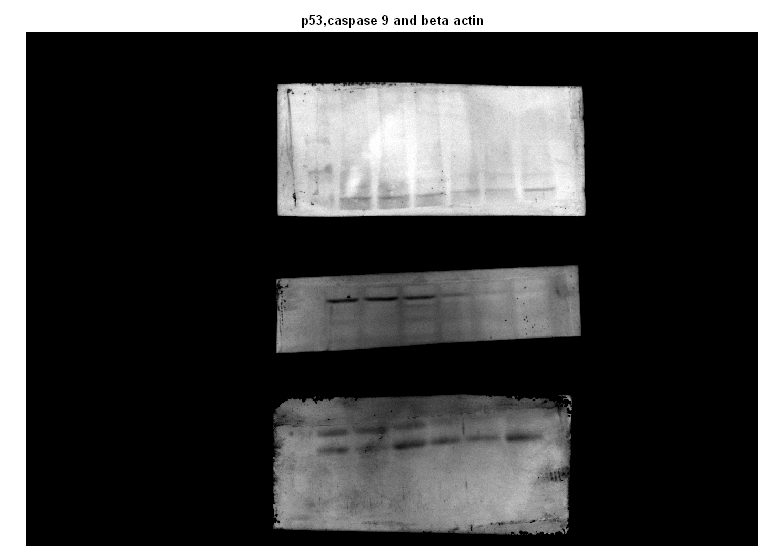
**

**p21**

**
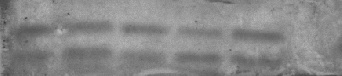
**

**β-actin**

**Figure S5. The full length blots or original images for Figure 9a**

**C 25 50 100 200**

**BS (μM)**


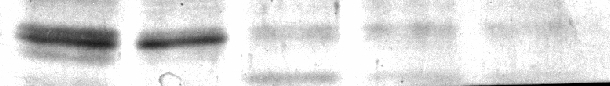


**Trx1**


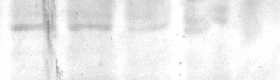


**TrxR1**


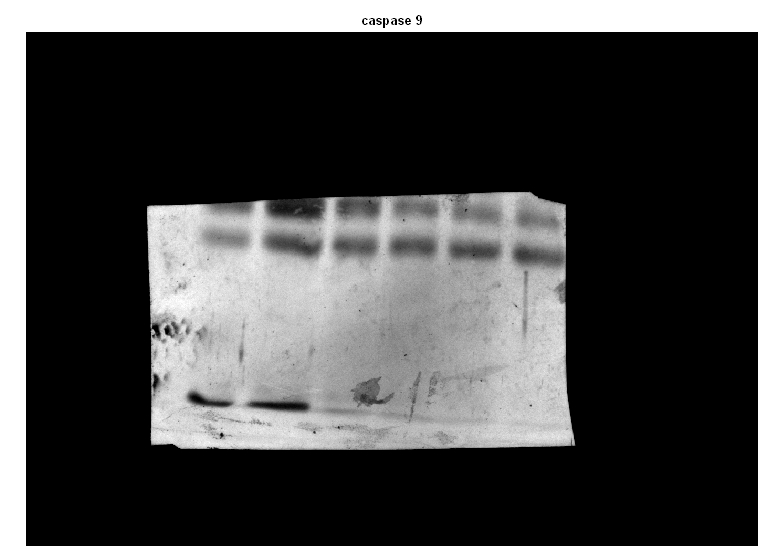


**β-Actin**


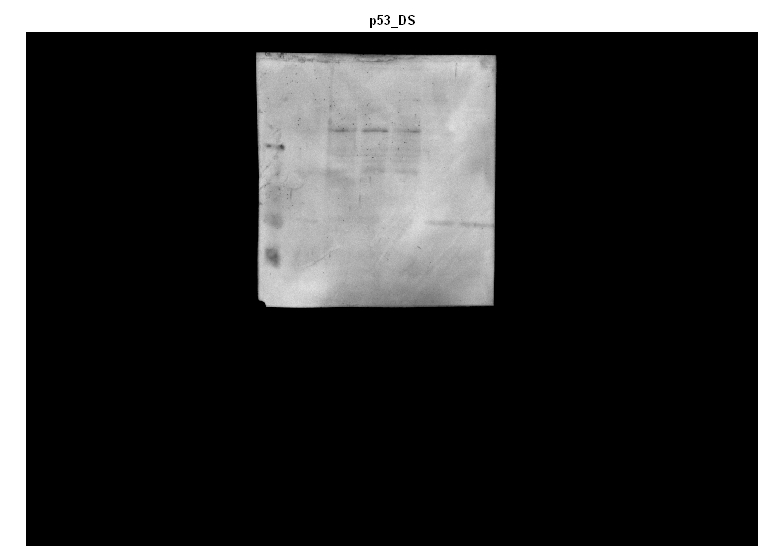


**TrxR1**


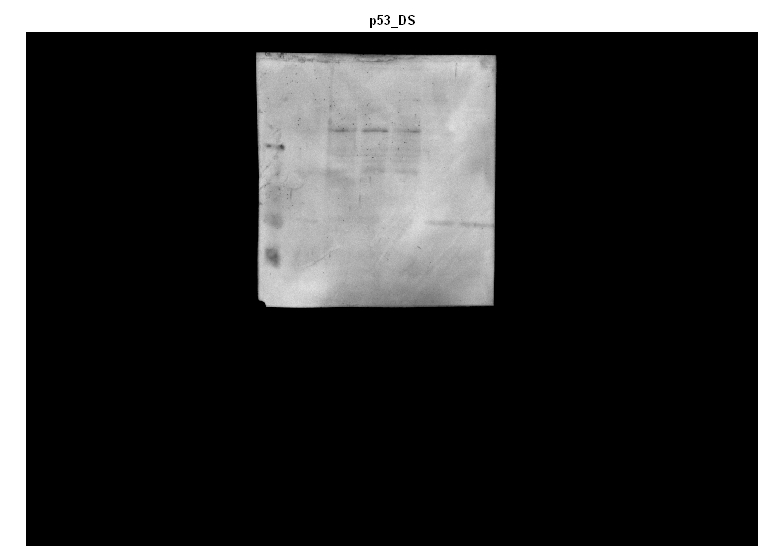


**Trx1**


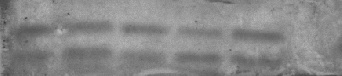


**β-actin**
